# Sorafenib—Drug Delivery Strategies in Primary Liver Cancer

**DOI:** 10.3390/jfb16040148

**Published:** 2025-04-21

**Authors:** Piotr Szyk, Beata Czarczynska-Goslinska, Marta Ziegler-Borowska, Igor Larrosa, Tomasz Goslinski

**Affiliations:** 1 Chair and Department of Chemical Technology of Drugs, Poznan University of Medical Sciences, Rokietnicka 3, 60-806 Poznan, Poland; 2 Doctoral School, Poznan University of Medical Sciences, Bukowska 70, 60-812 Poznan, Poland; 3 Chair and Department of Pharmaceutical Technology, Poznan University of Medical Sciences, Rokietnicka 3, 60-806 Poznan, Poland; bgoslinska@ump.edu.pl; 4 Department of Biomedical Chemistry and Polymer Science, Faculty of Chemistry, Nicolaus Copernicus University in Torun, Gagarina 7, 87-100 Torun, Poland; martaz@umk.pl; 5 Department of Chemistry, University of Manchester, Chemistry Building, Oxford Road, Manchester M13 9PL, UK; igor.larrosa@manchester.ac.uk

**Keywords:** drug delivery systems, nanoparticles, primary liver cancer, sorafenib, targeted therapy

## Abstract

Current primary liver cancer therapies, including sorafenib and transarterial chemoembolization, face significant limitations due to chemoresistance caused by impaired drug uptake, altered metabolism, and other genetic modulations. These challenges contribute to relapse rates of 50–80% within five years. The need for improved treatment strategies (adjuvant therapy, unsatisfactory enhanced permeability and retention (EPR) effect) has driven research into advanced drug delivery systems, including targeted nanoparticles, biomaterials, and combinatory approaches. Therefore, this review evaluates recent advancements in primary liver cancer pharmacotherapy, focusing on the potential of drug delivery systems for sorafenib and its derivatives. Approaches such as leveraging Kupffer cells for tumor migration or utilizing smaller NPs for inter-/intracellular delivery, address EPR limitations. Biomaterials and targeted therapies focusing on targeting have demonstrated effectiveness in increasing tumor-specific delivery, but clinical evidence remains limited. Combination therapies have emerged as an interesting solution to overcoming chemoresistance or to broadening therapeutic functionality. Biomimetic delivery systems, employing blood cells or exosomes, provide methods for targeting tumors, preventing metastasis, and strengthening immune responses. However, significant differences between preclinical models and human physiology remain a barrier to translating these findings into clinical success. Future research must focus on the development of adjuvant therapy and refining drug delivery systems to overcome the limitations of tumor heterogeneity and low drug accumulation.

## 1. Introduction

PLC originates from the tissue of the liver. There are two main types of PLC—hepatocellular carcinoma (HCC), affecting liver cells, and cholangiocarcinoma, affecting the bile ducts (including intrahepatic ICC and extrahepatic cancer). Less common PLCs are fibrolamellar carcinoma, hepatoblastoma, and mesenchymal liver cancers, such as angiosarcoma of the liver and epithelioid hemangioendothelioma [1].

The incidence rates of LC vary between and within countries, which indicates differences in exposure to risk factors. The indisputable cause of PLC is chronic infection with the hepatitis B and hepatitis C viruses (HBV and HCV), and the majority of PLC is attributed to the combined effects of persistent HBV or HCV infections. Other risk factors include aflatoxin exposure in diets, cigarette smoking, alcohol consumption, and oral contraceptives [2]. The incidence of HCC has been increasing since the 1980s and is now the fastest-growing cause of cancer-related death in the United States [3]. Its aggressiveness results from a transformation of the primary tumor cells into circulating tumor cells (CTCs) and metastasis at an early stage to distant organs through blood [4,5]. For that reason, early diagnostics of metastatic signs and effective blocking of HCC progression may contribute to a better prognosis for those patients. Unfortunately, usually, only abnormal symptoms induced by metastatic lesions or radiography allow for revealing advanced disease, missing the best time window for surgical resection [4]. CTCs activate platelets and form a microenvironment around them, which makes it more difficult for medications to reach them [5]. Moreover, neutrophils can directly adhere to CTCs and link tumor cells and the liver parenchyma, thus promoting extravasation and liver metastasis. Thus, CTC clusters with neutrophils anchor to the vascular endothelium for extravasation, which is mediated by a series of cell adhesion proteins, such as cadherin, integrin, and surface glycoprotein [6,7]. Therefore the effective elimination of CTCs is the key factor to preventing HCC metastasis [5]. For early diagnosis of HCC and tumor surveillance in patients receiving therapy CTCs, liquid biopsy may prove to be a useful tool [8]. However, detection of CTCs performed with conventional strategies is difficult due to inherently heterogeneous and dynamic expression of the epithelial cell adhesion molecule (EpCAM, CD326), as well as degradation of cytokeratins during the epithelial–mesenchymal transition, which contributes to their false negative detection [9].

The approach to liver cancer treatment is related to its stage, described by the Barcelona Clinic Liver Cancer (BCLC) staging system, concerning patient classification and treatment allocation. Patients at early stages (BCLC stage 0-A) can receive curative treatments such as resection, liver transplantation, or local ablation. Patients at the intermediate stage (BCLC stage B) with normal liver function could benefit from locoregional treatments such as transarterial chemoembolization (TACE), transarterial radioembolization (TARE), and stereotactic body radiation therapy (SBRT). Most HCC patients with disease progression after TACE or diagnosed with advanced-stage HCC (BCLC stage C) have to undergo systemic therapy, including chemotherapy, targeted therapy, and/or immunotherapy [10,11]. In such cases, tyrosine kinase inhibitors such as sorafenib or regorafenib and a combination of atezolizumab (anti-programmed death-ligand 1 -PD-L1- monoclonal antibody) and bevacizumab (antivascular endothelial growth factor monoclonal antibody) are mainly used, which may provide a median survival of below 2 years [8]. Patients at the end stage (BCLC stage D) receive palliative care [10,11].

Anti-PLC targeted drugs have been extensively researched due to their promising contribution to liver cancer treatment if surgical procedures are impossible or if the cancer has spread to other parts of the body. Precise localization of tumor cells facilitates and enhances drug accumulation. Targeted drugs exhibit the ability to selectively eliminate tumor cells while reducing damage to healthy tissues, which results from their enhanced aggregation at the lesion site [12]. Targeted drug delivery systems demonstrate many advantages over conventional drug formulations. They enhance drug delivery, improve drug stability, and prolong circulation time in vivo. Therefore, they are often applied in antitumor drugs and gene drugs with poor water solubility [13,14].

Although oral tablets or capsules are the most convenient and popular administration form for drugs to treat patients with liver cancer [15], their drawbacks are poor bioavailability and the necessity to increase drug doses to achieve the therapeutic effect. Long-term use of high drug doses poses a threat to health, leading to systemic toxicity. Moreover, the pH of the gastrointestinal tract affects drugs present in its environment and decreases its therapeutic efficacy [16].

Therefore, nanoformulations designed to deliver drugs to tumor cells in a targeted mode are a promising strategy to solve these shortcomings. Employment of specific ligands, antibodies, polysaccharides, and other materials to modify nanoparticles results in more selective targeting of tumor cells and leads to a reduction in drug doses required for treatment. Additionally, such novel formulations overcome the systemic toxicity and drug resistance caused by the long-term accumulation of active pharmaceutical ingredients in the body, which improves its antitumor ability [14].

Sorafenib is a first-line targeted drug for patients with advanced HCC, showing anti-angiogenic and antiproliferative effects on cancer cells [12,17], but with efficacy constrained by its off-target distribution, short circulation time in vivo, multi-drug resistance, and undesirable side effects. To overcome these shortcomings, researchers have been developing various delivery systems [18]. Passive targeting utilizes the disease physiology [19], and such delivery systems include carriers useful for improving solubility, increasing absorption, and contributing to a reduction in systemic toxicity such as liposomes, nanoemulsions, micelles, nanoparticles, nanocrystals, dendrimers, etc. Many benefits are also achieved by the application of active targeting delivery systems utilizing tailored surface coatings or conjugated ligands [20], such as ASGPR, FR, GPC3 protein receptor, LDLR, etc. [12]. Considering various physicochemical properties of the tumor environment, different exogenous stimulus responsiveness can also be used to respond to specific physicochemical conditions, such as pH, temperature, redox reactions, magnetic field, light, and ultrasound [12,21]. Last but not least, the combined treatment regimens of sorafenib by different drugs or treatment modalities (chemotherapy, immunotherapy, photodynamic therapy, photothermal therapy) have shown favorable efficacy in the fight against cancer due to a wider treatment range, drug dose reduction, and, to some extent, reverse multidrug resistance [22]. Drug delivery systems hold promising prospects for cancer therapeutics in the future.

The aim of this review is to critically evaluate recent progress in sorafenib delivery for PLC, with an emphasis on targeted drug delivery systems and strategies to overcome chemoresistance. Through analysis of targeted, biomimetic, and combinatory NPs, this work highlights novel approaches to improve the efficacy of sorafenib and related treatments. Moreover, it evaluates receptors utilized in targeting and identifies translational challenges between human and animal models. Apart from the assessment of current strategies and their limitations, this review identifies key areas for future research.

## 2. Liver Cancer Treatment

### 2.1. Current Approach

According to the National Cancer Institute, various types of treatment for localized liver cancer can be proposed. For lesions smaller than 1 cm, follow-up is recommended every 3 months. Surgical removal of the part of the liver affected by cancer, along with some of the healthy tissue around it, is also conducted. In the case of a liver transplant, the entire liver is replaced with a healthy donated organ. Another treatment approach that is very helpful but less likely to cure cancer is ablation, which is suitable for tumors no larger than 3 cm across and localized away from major blood vessels, the diaphragm, or major bile ducts [23]. This technique destroys liver tumors and some of the normal tissue around the tumor. It is considered a potential first-line treatment in many patients with small hepatocellular carcinomas or benign liver tumors. During tumor ablation, thermal energy is used to heat or cool tissue to cytotoxic levels. Less than −40 °C is used for cryoablation, and more than 60 °C for microwave ablation and radiofrequency ablation. Nonthermal techniques include irreversible electroporation. During the procedure, electrical pulses are sent through an electrode placed in a tumor to kill cancer cells [23,24]. Also, percutaneous ethanol injection can be applied directly to a tumor to kill cancer cells [10]. The next type of therapy for patients with liver cancer whose tumor has not spread outside the liver is embolization. It is used for those who cannot be subjected to surgery or ablation therapy. The procedure consists of the injection of substances directly into the hepatic artery to block or reduce the blood flow to the liver tumor. The tumor will not continue to grow because of the lack of oxygen and nutrients. There are two types of embolization—transarterial embolization (TAE) and transarterial chemoembolization (TACE). The first one concerns a small incision made in the inner thigh followed by a catheter insertion. After threading it up into the hepatic artery, a required substance is injected. An anticancer drug is administered in the TACE procedure, also called chemoembolization. The drug can be attached to tiny beads, which are injected into the hepatic artery, or it can be injected through a catheter into the hepatic artery before injecting the substance aimed at blocking the hepatic artery [23]. Conventional TACE using lipiodol is a pivotal therapeutic modality for HCC. Lipiodol derived from poppy seeds has many advantages. It is radiopaque, reaches tumor tissues, and remains within the target area for an extended period, thereby assisting in the transient embolization of the tumor microcirculation during TACE [25]. Lipiodol does not accumulate in the Kupffer cells of the normal liver but rather in tumor cells, which makes it an imaging marker of tumor necrosis after TACE [26]. Delivery of exogenous lipiodol after resection has also shown increased recurrence-free survival in HCC patients [27]. Heterogeneous lipiodol accumulation should be a warning for recurrence in previously treated HCC nodules. Recognizing lipiodol accumulation patterns could contribute to prognostic assessment and early intervention strategies to potentially enhance patient survival following TACE [25,28]. Lipiodol-based TACE is typically used as the gold standard for comparative studies with other TACE procedures (using drug-eluting beads), radioembolization, or targeted therapy (sorafenib) in patients with intermediate or advanced HCC [29]. Targeted therapy means the application of drugs to attack specific cancer cells. Such therapies include the treatment of advanced liver cancer with sorafenib, bevacizumab, cabozantinib, lenvatinib, ramucirumab, and regorafenib [23]. In immunotherapy, a patient’s immune system is used to attack cancer cells. Substances made by the body or chemical drugs are used to boost, direct, or restore the body’s natural defenses against cancer. Also, immune checkpoint inhibitors may be used for treatment. Last but not least is radiation therapy directed at the area with cancer [23].

### 2.2. Sorafenib in Liver Cancers

Sorafenib is classified as a second-class drug in the Biopharmaceutical Classification System (BCS) due to its low aqueous solubility (~2 ng/mL) [30] and high permeability of the gastrointestinal membrane. Poor solubility over a wide pH range (1.2–7.4) and slow dissolution in the gastrointestinal tract (GIT) lead to its low oral bioavailability (about 8.43%) and large inter-subject fluctuations [31,32]. Sorafenib is also very lipophilic (LogP = 3.8) and has a strong crystal lattice (Tm = 205 °C) [33]. Sorafenib is available in a base, as well as hydrochloride, hydrobromide, methylsulfonate, sulfate, hemi-tosylate, and tosylate forms [34]. Its commercial formulation contains sorafenib tosylate, with improved but still poor aqueous solubility, affecting absorption through GIT [31]. The oral tablet Nexavar, with crystalline sorafenib tosylate, provides an oral bioavailability of 38–49% [30,33]. Poor solubility, rapid metabolism, and low bioavailability contribute to the limitation of the clinical applications of sorafenib, and these issues drive research on new formulations to improve drug targeting and therapeutic efficacy in HCC. A variety of approaches have been used, such as parenteral administration, hydrophilic and water-soluble polymers, lipid-based formulations, silica and metal nanoparticles (NPs), solid dispersion technology, a self-micro-emulsifying drug delivery system, nanocrystals, and the addition of surfactants [31,35,36].

**Figure 1:**
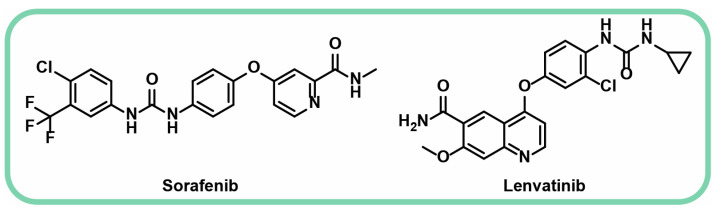
Sorafenib and lenvatinib.

Sorafenib, as a multikinase inhibitor, is considered an effective chemotherapeutic agent against various types of tumors [18]. It (Figure 1) is a first-line treatment for prolonging life of patients who have either failed transarterial chemoembolization or who suffer from more advanced HCC [37]. It has shown effectivity not only against HCC but also against renal-cell carcinoma [38,39] and thyroid cancer [31,40,41]. The use of sorafenib is now being expanded to treat acute myeloid leukemia, desmoid tumors, and metastatic melanoma [42].

**Figure 2:**
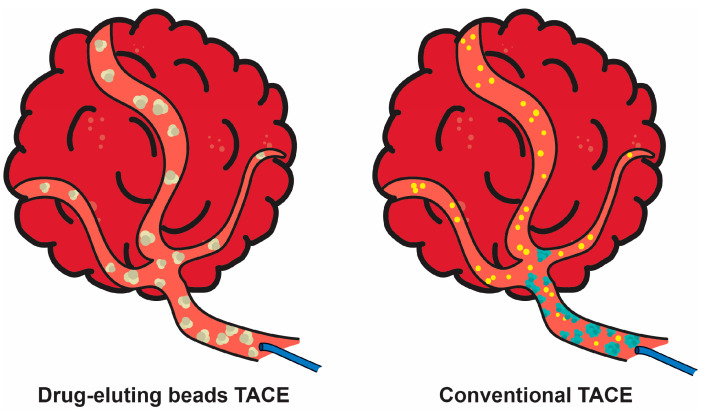
Comparison of conventional and drug-eluting TACE beads.

Sorafenib is the first of the two approved targeted agents for HCC (with lenvatinib (Figure 1) being the second one) [43]. It is administered as tablets orally, with a daily recommended dose of 400 mg [31,44]. Sorafenib improves survival, resulting in slower cancer progression over time [37]. As an anti-angiogenic VEGF inhibitor, it has recently been added to TACE to increase the overall survival and time to progression in HCC patients [45]. Conventional TACE, widely considered the standard of care for treating unresectable HCC, involves intra-arterial administration of chemotherapeutic agents along with lipiodol, followed by injection of embolizing agents like gelatin sponge particles to induce necrosis of the tumor tissue [45,46]. The second approach utilizes microspheres that simultaneously release the drug and facilitate embolization [47] (Figure 2).

**Figure 3:**
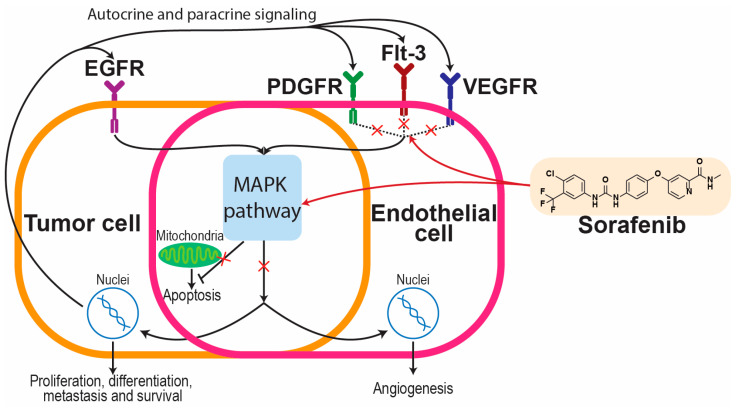
Mechanism of action of sorafenib (based on [54,55,56,57]). MAPK—mitogen-activated protein kinase; EGFR—epidermal growth factor receptor; PDGFR—platelet-derived growth factor receptor; VEGFR—vascular endothelial growth factor receptor; Flt-3—fms-like tyrosine kinase 3.

It is an orally administered multikinase inhibitor with activity against RAF (rapidly accelerated fibrosarcoma) kinases (CRAF, BRAF, mutant BRAF), vascular endothelial growth factor receptor (VEGFR-1, VEGFR-2, VEGFR-3), platelet-derived growth factor receptor (PDGFR-β), and several other kinases [37,40,48]. This active pharmaceutical ingredient also increases the apoptosis rate in many types of cancer [31]. Frequent oncogenic mutations have been identified in MAPK (mitogen-activated protein kinase) signaling pathway components. Therefore, the MAPK pathway contributes to human cancer initiation, in particular the RAF component. The mutation in the RAF component causes auto-activation of the MAPK signaling pathway RAS-RAF-MEK-ERK (RAS—rat sarcoma; RAF—rapidly accelerated fibrosarcoma; MEK—mitogen-activated protein kinase; ERK—extracellular signal-regulated kinase), which induces uncontrolled cell growth and proliferation [49]. The activation of the MAPK pathway can also be caused by the activation of RAS (rat sarcoma) proteins and lead to resistance to apoptosis-inducing drugs [50,51]. Blood vessel formation around the developing cancer cells can be prevented by targeted anti-angiogenic therapy (AAT), as vascular endothelial growth factor (VEGF) and VEGFRs play significant roles not only in physiological but also in most pathological angiogenesis [52]. Receptor tyrosine kinases (RTKs) are key regulatory signaling proteins that control cancer cell growth and metastasis. Over the past two decades, several molecules targeting RTKs have been applied as a first- or second-line therapy for different types of cancer [53]. The mechanism of action of sorafenib is presented in Figure 3.

Its associated toxicity is easily managed and includes a hand–foot skin reaction, diarrhea, hypertension, rash, fatigue, abdominal pain, and nausea [58]. Moreover, bilirubin elevation, thrombocytopenia, aspartate aminotransferase (AST) elevation, anorexia, and alopecia are often reported. All these disorders are reported as dose-limiting toxicities [59].

It is mainly metabolized in the liver via an oxidative pathway and glucuronidation via uridine diphosphate glucuronyl transferase 1A9 (UGT1A9) [40,60,61]. Oxidative metabolism of sorafenib is initially mediated by P450 3A4 (CYP3A4). Therefore, targeting CYP3A4 may help increase the sensitivity of HCC cells to chemotherapeutic agents [62]. In the gastrointestinal tract, bacterial glucuronidase enzymes may hydrolyze glucuronide conjugates, leading to the reabsorption of unconjugated drug. Sorafenib glucuronides can be hydrolyzed in the gastrointestinal tract by β-glucuronidase, and unconjugated drug can be reabsorbed [63]. Metabolites of sorafenib have been excreted mainly in feces and urine [64]. Sorafenib has an elimination half-life ranging from 25 to 48 h [65].

There have been many reports on the efficiency of sorafenib in prolonging the overall survival of HCC patients in combination with TACE [45,66,67,68,69]. Moreover, sorafenib also increases the average interval and frequency of TACE, thereby increasing lipiodol deposition and improving the antitumor effect [45,70]. However, Zheng et al. [45] observed no significant impact of lipiodol deposition on the survival benefits exerted by the synergistic combination.

## 3. Primary Liver Cancer Targeting

The conventional chemotherapeutics are nonselective and can also damage healthy tissues, contributing to adverse effects. Additionally, due to the poor bioaccessibility of these drugs to tumor tissues, higher doses are required, causing elevated toxicity in normal cells and an increased incidence of multiple drug resistance. Therefore, it is necessary to develop chemotherapeutics that can either passively or actively target cancerous cells, thus reducing adverse side effects and improving therapeutic efficacy [71]. Targeted therapy consists in blocking signaling pathways that promote cancer cell growth in the aftermath of identifying and binding to specific receptors on the surface of liver cancer cells, or to specific subcellular receptors. Therefore, tumor cell proliferation, metastasis, and angiogenesis are inhibited. Recently, more and more studies are focusing on the design of nanoparticle-based drug delivery systems for targeted cancer therapy because of their ability to precisely deliver therapeutic agents to tumor sites [72,73]. For better understanding of the interaction process between NP carriers and cancer cells, the targeting mechanisms can be broadly classified as either passive or active [74]. Passive targeting is a strategy accomplished by integrating the therapeutic agent into nanoparticles with specific physicochemical properties (size, charge, etc.) that accumulate preferentially in the neoplastic tissues as a result of the enhanced permeability and retention (EPR) phenomenon and specific characteristics of the tumor microenvironment [75,76]. For active targeting, surface-modified nanocarriers or specific ligands such as peptides, proteins, antibodies, aptamers, or folic acid are used to specifically bind to overexpressed receptors on tumor cells [18,73]. Although active targeting exhibits much higher specificity than passive targeting, it can be potentially immunogenic. Moreover, the active targeting approach is more expensive for developing a formulation due to the high cost of ligands, unlike the relatively simple and cheaper passive targeting [73].

### 3.1. Passive Accumulation

**Figure 4:**
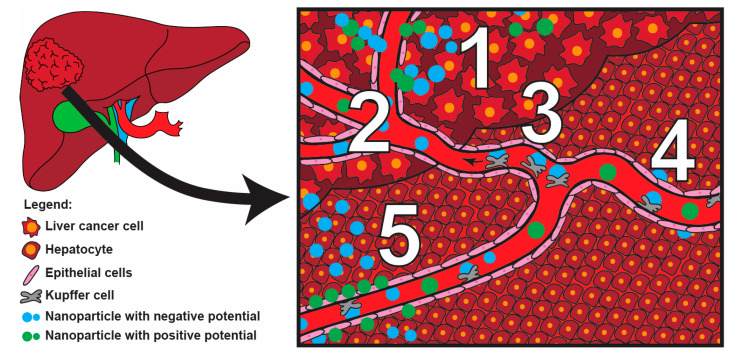
PLC passive targeting. 1—EPR effect: NPs penetrate through the pores; 2—heterogeneity in vasculature; 3—the tumor-homing ability of macrophages; 4—NPs with negative potential are uptaken by Kupffer cells; 5—small NPs permeate through the vessel to liver tissue, and those with positive potential are uptaken by hepatocytes, while those with negative potential penetrate the tissue.

One of the significant advantages of utilizing NPs for the treatment of PLC is their natural tendency to accumulate in the liver, even in the absence of specific ligands [77,78,79,80,81,82]. This effect can be further enhanced by selecting NPs with hydrophobic surfaces, which promote binding to plasma proteins and facilitate liver uptake [83,84,85,86]. However, it should be noted that these NPs are often internalized by Kupffer cells (Figure 4) and subsequently excreted into the intestines, which is undesirable for drug delivery as it limits the amount of the therapeutic agent reaching the target site [86]. An alternative mechanism that could enhance tumor accumulation involves the tumor-homing ability of Kupffer cells (Figure 4), which is based on their migration to tumor tissues [83,84]. While promising, this mechanism requires further investigation to fully elucidate its potential and optimize its application. The size and surface charge of NPs are critical factors influencing their cellular uptake. Positively charged NPs are preferentially internalized by hepatocytes (Figure 4), whereas negatively charged NPs are more likely to be taken up by Kupffer and endothelial cells [87]. To further increase hepatocyte internalization, NPs with sizes smaller than 150–200 nm can be designed, as these are capable of crossing capillaries through small fenestrations and interacting directly with hepatocytes [88]. Ideally, NPs should accumulate in tumor tissues. Therefore, strategies to enhance tissue penetration and uptake by PLC cells should be prioritized when significant accumulation occurs in the liver. Alternatively, ensuring efficient clearance of NPs by hepatocytes following uptake is critical to mitigate the risk of hepatotoxicity [88,89].

Another important mechanism of passive NP accumulation in tumors is the enhanced permeability and retention (EPR) effect, which is a hallmark of cancer tissues. This phenomenon arises from the discontinuous vasculature structure of tumors and the absence of functional lymphatic drainage. These characteristics facilitate the vascular permeation of NPs into tumor tissues and their subsequent retention (Figure 4). The EPR effect has been demonstrated in animal models of PLC [77,78,79,80,81,82], with small-sized NPs (~12 nm) being particularly effective. NPs of this size achieve significant tumor accumulation while minimizing nonspecific uptake in major organs. However, NPs smaller than 6 nm are subject to rapid renal clearance, which reduces their therapeutic utility [90,91]. Despite these promising findings in preclinical models, clinical trials indicate that drug delivery systems (DDSs) relying solely on the EPR effect are often insufficient. These effects are collected in Table 1. Tumor progression in humans differs substantially from that in rodent models in terms of both tumor biology and therapeutic scheduling. Human tumors typically accumulate mutations over a prolonged period prior to the main growth, resulting in greater heterogeneity (Figure 4) and fewer permeable surfaces in the tumor vasculature [89,92,93]. These differences diminish the effectiveness of the EPR-based strategy, highlighting the need for more sophisticated approaches.

One such strategy is targeted therapy, which exploits specific receptors on cancer cells to enhance NP delivery. This approach has demonstrated approximately 1.5 times higher delivery efficiency than non-targeted systems [89,94]. In addition, incorporating additives to improve DDS penetration into tumor tissues is under investigation [93]. These advanced methodologies not only enhance drug delivery efficiency but also hold promise for overcoming chemoresistance mechanisms commonly observed in PLC [95,96].

### 3.2. Receptor-Targeted Accumulation

**Figure 5:**
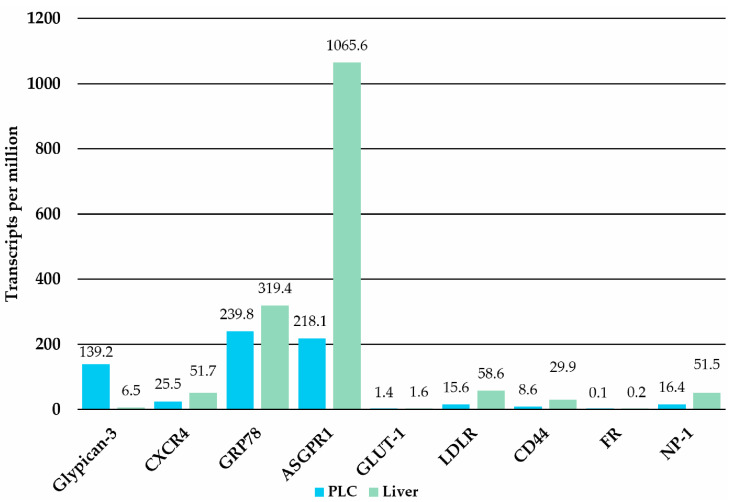
Comparison of receptor expression between liver and PLC, based on Proteinatlas.org [97].

Targeting specific receptors and proteins overexpressed in PLC offers a promising approach for advancing diagnosis and treatment. This discussion delves into key molecular targets, including glypican-3, CXCR4, GRP78, ASGPR1, GLUT-1, LDLR, CD44, FR, and NP-1 (characterized below), emphasizing their expression patterns, therapeutic potential, and associated challenges. Each section highlights ligands for these receptors, such as antibodies, peptides, small chemical entities, and their connection to NP-based DDSs. The analysis also explores limitations, such as off-target effects and variable expression levels (Figure 5), while identifying opportunities for personalized therapies. In addition, these receptors are compared in Table 2. For example, the median expression of these receptors in patients is often lower in the cancer tissue than in the liver or other organs, except for GPC3 [97]. However, there are populations with overexpression, and consequently, there are also commercially available PLC cell lines with these features that undergo evaluation [98,99,100]. Moreover, some of the targeted therapies benefit from overexpression of the receptors in the liver, such as ASGPR, which leads to the accumulation of targeted DDSs in the liver [101]. By examining these molecular pathways, this overview provides insights into the strategies for combating PLC through receptor-specific targeting [97].

#### 3.2.1. Glypican-3

Glypican-3 (GPC3) is a membrane-bound proteoglycan that is highly expressed in the majority of HCC cases, but it is sparsely present in healthy liver cells. This selective expression makes GPC3 a promising candidate for both the treatment and diagnosis of PLC [150]. Proteins such as GPC3 are classified as oncofetal antigens due to their expression during fetal development and cancer progression [102]. Evidence suggests that GPC3 expression is specific to PLC (Figure 5), with minimal expression in other tissues except for the placenta [103], rendering its therapeutic targeting unsuitable during pregnancy [151]. Clinically, GPC3 has been successfully employed as a serological biomarker for cancer detection. When combined with alpha-fetoprotein measurements, the sensitivity of diagnostic tests improves significantly, as these proteins are often expressed mutually exclusively in HCC cases [152]. Current ligands for GPC3 include the L5 peptide (Arg-Leu-Asn-Val-Gly-Gly-Thr-Tyr-Phe-Leu-Thr-Thr-Arg-Gln) and a truncated derivative containing the last seven amino acids [104,105], recombinant human GPC3 core protein [106], antibody constructs, and aptamers. Peptides offer advantages such as ease of synthesis, small molecular size, and low immunogenicity but are susceptible to enzymatic degradation before reaching the target site. In contrast, antibodies, while more challenging and costly to manufacture, provide superior specificity, sensitivity, and a longer half-life. Aptamers, short nucleotide sequences ranging from 15 to 40 bases, are emerging biomolecules primarily studied as potential imaging agents for GPC3 [107]. Natural ligands of GPC3 include growth factors, cytokines, and chemokines [153], but no small-molecule chemical entities have been reported as potent ligands for this receptor. The therapeutic potential of targeting GPC3 has been clinically demonstrated, as outlined by Su et al. in their discussion of sorafenib, detailed elsewhere in this review [154]. The relative expression levels of GPC3 in various PLC cell lines are as follows: Li-7 < Huh7 < HepG2 [155].

#### 3.2.2. CXCR4

**Figure 6:**
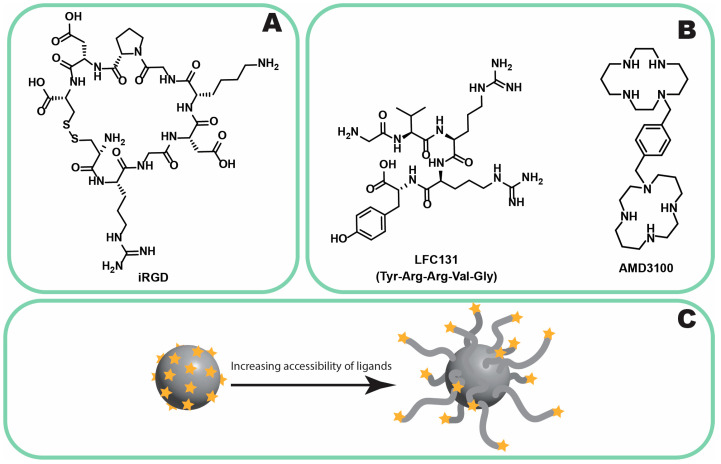
(**A**) Structure of iRGD; (**B**) antagonists of CXCR4 applied as targeting moieties for primary liver cancers, (**C**) increasing accessibility of ligands.

C-X-C motif chemokine receptor 4 (CXCR4) is a protein that plays a pivotal role in both cancer progression and viral diseases [156]. In PLC, CXCR4 overexpression has been linked to increased tumor metastasis, reduced survival rates [157], and resistance to sorafenib treatment [158]. Consequently, CXCR4 has become a prominent therapeutic target in various cancer types, with ligands including both small-molecule chemical entities and peptides [156,159]. Cordycepin, a compound derived from fungi, has been shown to inhibit the migration and invasion of PLC cells by downregulating CXCR4 expression [112]. Since 2024, specific ligands for CXCR4 in PLC have included the LFC131 peptide and AMD3100 (Figure 6B) [109,110,111]. Notably, Zheng et al. demonstrated that LFC131 effectively targets PLC cells with high CXCR4 expression, such as the SMMC-7721 cell line, but not in cells with lower CXCR4 expression, such as Huh7 [111]. The clinical utility of CXCR4 as a therapeutic target in PLC is, however, subject to debate. This is primarily due to its higher baseline expression in several normal tissues, particularly in bone marrow and lymphoid tissues, compared to its median expression in PLC (Figure 5) [108]. Such widespread expression could pose challenges for achieving tumor specificity and minimizing off-target effects, thereby complicating its application in clinical settings.

#### 3.2.3. Neuropilin-1

Neuropilin-1 is a membrane protein expressed by tumor and endothelial cells, playing a key role in angiogenesis and cell migration [113,114]. While the overexpression of neuropilin-1 in PLC remains debatable [115], several studies have reported the effectiveness of targeting this receptor in the disease. As of 2024, the cyclic peptide iRGD (Figure 6A) has been employed for neuropilin-1 targeting [116,117,118]. The advantages and limitations of iRGD were reviewed by Nikitovic et al. [114]. iRGD can be used either independently or conjugated to DDSs, with both strategies demonstrating efficacy and enhanced drug penetration/uptake in cancer tissues [116,117,118]. Notably, iRGD-based DDSs have progressed to the first and second phases of clinical trials and show promise in the treatment of pancreatic cancer [119,120,121]. However, the evidence supporting its application in PLC treatment remains limited.

#### 3.2.4. GRP78

GRP78, also known as the 78 kDa glucose-regulated protein, is a heat shock protein that is primarily located in the endoplasmic reticulum and, under specific circumstances, can migrate to the cell membrane in cancer cells. This protein is particularly overexpressed in drug-resistant cancer cells, including PLC [122]. Several ligands are known to bind to GRP78, such as the arginine–glycine–aspartic (RGD) peptide and SP94. However, SP94 appears to be the most effective ligand for targeting PLC [123]. SP94 is a peptide with the sequence SFSIIHTPILPL, and it demonstrates preferential binding to hepatocellular carcinoma (HCC) cells, such as Huh7 and HepG2, rather than to normal hepatocytes, immune cells, or endothelial cells [160,161]. To optimize SP94’s efficacy in vivo and enhance its accumulation at tumor sites, it must be conjugated to a DDS [162] rather than used in any other way. Several factors, including the degree of modification, the size of the linker, and the site of connection to the ligand, influence the binding affinity of SP94-modified DDSs to PLC. Notably, C-terminal conjugation should be avoided, as it reduces SP94’s binding affinity to HCC cells. A higher degree of modification is desirable, but it is essential to ensure the exposure of individual peptides on the DDS surface. Longer linkers are recommended to create adequate spacing between ligands, distancing them from the surface and improving accessibility for receptor binding (Figure 6C) [161]. However, the hydrophobic nature of SP94 may increase opsonization and uptake by immune cells such as Kupffer cells. To mitigate this, the incorporation of polyethylene oxide (PEO) chains or the use of more hydrophilic ligands such as saccharides may be beneficial [124]. The first therapeutic evaluations of SP94-modified NPs reported enhanced effectiveness compared to non-targeted counterparts. These targeted DDSs demonstrated increased tumor accumulation, tumor growth regression, and an improved selectivity index (SI) [163]. The data suggest that these effects are primarily mediated by SP94 itself, as the differences in tumoral accumulation between non-targeted and SP94-targeted DDSs are significant [164,165]. Various GRP78-targeted DDSs for PLC have been developed, employing diverse materials, including exosomes [77,166], lipids (e.g., liposomes) [167], non-organic and semi-organic materials (e.g., organic silica, metal-organic frameworks [MOFs], multilayered NPs [116,164,165,168], polymers [169,170], and even viral compartments [123].

#### 3.2.5. ASGPR

**Figure 7:**
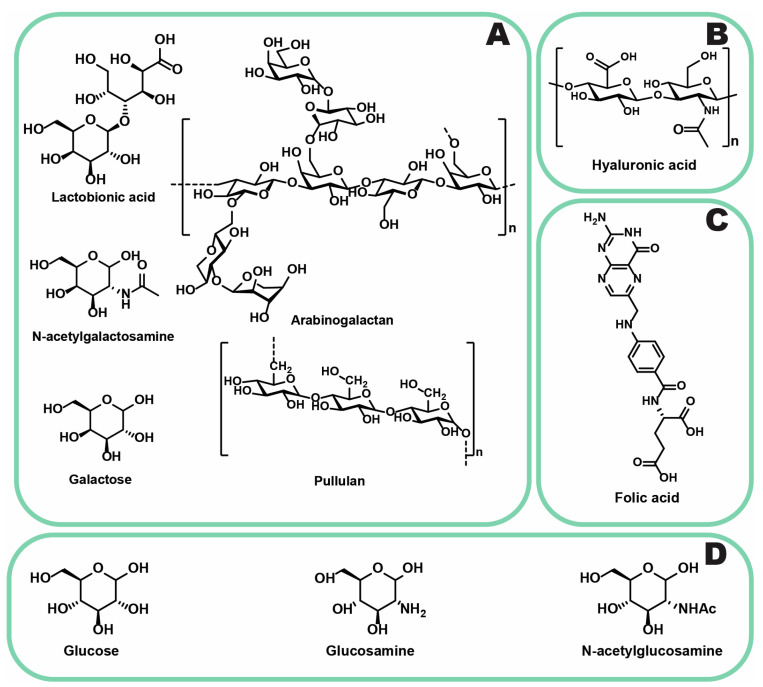
(**A**) Ligands of ASGPRs; (**B**) ligand of CD44; (**C**) ligand of folate receptor; (**D**) ligands of GLUT-1.

Asialoglycoprotein receptors (ASGPRs) are among the most extensively studied targets for liver-specific DDSs and receptor-mediated uptake. The primary physiological role of ASGPRs is to capture glycoconjugates that are potentially hazardous to the organism, facilitating their removal. In Japan, these receptors have been clinically utilized to assess liver functional mass. ASGPRs are predominantly expressed on liver cells and hepatocellular tumors, making them a promising target for the development of novel DDS and imaging agents. Nevertheless, some sources indicate rather lower expression of ASGPRs in cancer cells [125]. Ligands for this receptor include galactose, N-acetylgalactosamine [126], and their derivatives, such as lactobionic acid [171], polymers (e.g., pullulan [127], arabinogalactan [128]), and biomolecules (glycoproteins and their glycans [172]) (Figure 7A). In contrast to peptides, these saccharides are more polar and undergo smaller opsonization [124]. Both synthetic and natural ligands have been evaluated, providing valuable insights for the rational design of ASGPR ligands, including the selection of sugar isomers, galactose moiety density, branching patterns, valency, and spacer configuration. These findings were comprehensively reviewed by D’Souza and Devaraja [101]. A key challenge in utilizing ASGPRs lies in the variability of its expression among liver cancers. This necessitates the development of advanced screening assays to enable the personalized selection of targeted delivery therapies for individual patients [129]. Variability in ASGPR expression is also evident in commonly used cell lines; HepG2 and HepAD38 cells exhibit high receptor density, whereas Huh7 and HuH-6 cells show significantly lower expression [173].

#### 3.2.6. GLUT-1

Glucose transporter 1 (GLUT1) is a membrane protein widely expressed in various cell types, facilitating glucose uptake. Cancer cells, including PLC cells, exhibit an elevated demand for glucose, thus contributing to the upregulation of GLUT1 expression. Specifically, PLC cells are characterized by increased GLUT1 expression, whereas GLUT2 expression is typically reduced. The heightened glucose requirement is further intensified in hypoxic cells, where energy deprivation triggers adaptive responses. Hypoxic regions are known as the origins of chemoresistant and treatment-surviving cell populations, which often exhibit overexpressed GLUT1 transporters [130,131]. Such cells have been observed to arise during TACE, with evidence indicating enhanced proliferation and division among them [131,135]. GLUT1 overexpression holds potential both as a prognostic biomarker and as a molecular target for therapy. Strategies targeting GLUT1 include suppressing its expression or modulating its function. Notably, inhibiting GLUT transporters can sensitize liver tumor-initiating cells to sorafenib treatment [131,174]. The role of GLUT1 in PLC has been reviewed by Amann and Hellerbrand [135]. However, targeting GLUT1 has notable disadvantages. Molecules with GLUT affinity have been reported to cross the blood–brain barrier (BBB), posing risks in the context of GLUT deficiency syndrome [135]. An alternative therapeutic approach leverages GLUT1 as a transporter for DDSs. In this context, NPs modified with ligands such as glucosamine [133], N-acetylglucosamine [134], and glucose [175,176,177] (Figure 7D) can be developed. These ligand-functionalized NPs demonstrate improved therapeutic efficacy compared to non-targeted systems [133].

#### 3.2.7. LDL Receptor

The low-density lipoprotein receptor (LDLR) is a membrane protein that facilitates the cellular uptake of lipids embedded within lipoprotein particles from the extracellular fluid. A key component of lipoproteins is apolipoproteins (Apos), which mediate receptor binding and uptake [178,179]. For liver-targeted applications, Apo-B and -E have been employed [80,136]. Although LDLR expression is detectable in nearly all human tissues, it is markedly elevated in cancer and liver cells, making it a promising target for PLC therapy [136,137,138,139]. Despite its potential, the mechanisms governing LDLR-mediated liver targeting—such as the role of ligands, their conjugation to DDSs, and patterns of tissue-specific accumulation—remain incompletely understood. Two strategies can be employed for LDLR targeting: utilizing endogenous or exogenous Apos. During opsonization, NPs become enriched with endogenous Apos and are subsequently internalized by macrophages, which naturally accumulate in tumor tissue due to their tumor-homing ability [83,84]. This phenomenon was described by Li et al. [139] and Sebastiani et al. [85], who suggested that Apo-opsonized NPs are directly internalized through LDLR. For LDLR-targeted PLC therapies, the DDS matrix appears to play a critical role. Lipid-based carriers with hydrophobic surfaces are particularly advantageous [85]. Dalhaimer et al. provide a review of this topic [180]. However, functionalization with Apos poses challenges, such as increased permeability across the BBB, which is undesirable in PLC treatments [140].

#### 3.2.8. CD44

CD44 is a cell surface antigen expressed on most cells and is involved in various biological functions, such as cell adhesion and hyaluronate degradation [181,182]. In PLC, overall CD44 expression is typically low to moderate, with several other tissues exhibiting higher expression levels. However, certain PLC cell lines, such as SK-HEP-1 [145], demonstrate high CD44 overexpression, which is correlated with increased tumor invasiveness and recurrence rates [183]. Notably, PLC stem cells are characterized by high CD44 expression, making this receptor a compelling target for therapeutic strategies aimed at achieving tumor regression and reducing recurrence through the eradication of cancer stem cells [141,142,143,144]. Hyaluronic acid (HA) (Figure 7B) is a well-known ligand for CD44, and its use in DDSs has shown improved therapeutic efficacy compared to non-targeted approaches for PLC treatment [81,133].

#### 3.2.9. Folate Receptor

Folate receptors are responsible for the cellular uptake of folic acid (Figure 7C) [148]. Several types of cancer, including breast, kidney, and lung cancers, exhibit significant overexpression of this receptor. In contrast, PLC is typically associated with normal levels of folate receptor expression [146,147]. This targeted approach is not particularly promising for PLC treatment and may pose challenges such as the potential crossing of the BBB by the DDS and inadvertent targeting of the choroid plexus [149]. However, improved therapeutic outcomes have been observed in vivo and in vitro with folate receptor-targeted DDSs, particularly when using SMMC-7721 or BEL-7402 cell lines, which exhibit high folate receptor expression [98,99,100].

## 4. Sorafenib and Its Derivatives in PLC

**Figure 8:**
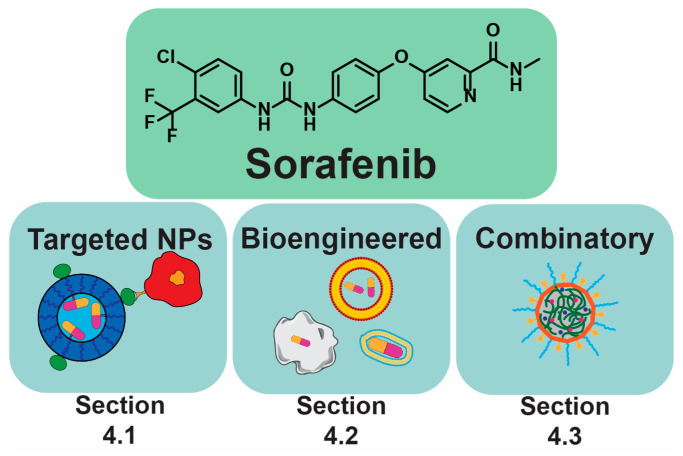
Approaches for the delivery of sorafenib described in the following sections.

Sorafenib has been the most extensively researched drug in the field of DDSs for PLC. A drug delivery system is a set of components working together as parts of a mechanism allowing the delivery of a drug to a target site of pharmaceutical action [184]. It includes technologies that tune drug preparation, route of administration, site targeting, metabolism, and toxicity. As a systemic treatment, it exhibits limited targeting specificity against PLC [154,185]. Recently, there has been a growing interest in nano- and micro-scale DDSs across various fields, including medicine and even agriculture [186]. This innovative approach has been clinically tested for treating PLC in humans. Wang et al. [187] reported on Callispheres^®^, drug-eluting beads composed of poly(vinyl alcohol), for the delivery of sorafenib during TACE. Preclinical evaluations in mice showed that Callispheres prolong the drug’s half-life, maintain stability in biological environments such as serum and liver cancer cells, and reduce the required dosage without compromising therapeutic efficacy. Clinically, this method significantly reduced tumor volume in the short term and achieved a higher rate of complete remission in PLC compared to standard TACE with sorafenib. Furthermore, reduced liver damage was observed. A two-year follow-up revealed that patients treated with Callispheres had better survival rates, fewer relapses, and reduced disease progression [187]. Another clinical trial, documented by Liu et al. [188], examined Callispheres loaded with regorafenib. The TACE using these beads outperformed standard TACE therapy for PLC, with significantly higher objective response rates (73.3% vs. 10.5%) and disease control rates (86.7% vs. 47.4%) based on mRECIST-modified criteria. Additionally, the median progression-free survival for the novel therapy was nine months compared to six months for the standard approach. Importantly, there was no statistically significant difference in the incidence of regorafenib-related adverse reactions between the two groups [188]. These clinical trials underscore the potential of micro- and nano-drug delivery systems to become standard therapeutic approaches. In the following text, three different ideas for NPs will be described (Figure 8). These include targeted NPs, the main feature of which is the presence of specific ligands in the composition; bioengineered NPs, with biological parts in the structure that aim for biomimetics; and combinatory NPs, which include additional therapeutic/imaging agents inside. All the mentioned approaches are focused on increasing drug efficiency, either by active targeting, or by passive or biomimetic accumulation in cancer cells. Moreover, the employment of additional therapeutic factors—other drugs (doxorubicin), siRNA, ferric oxides, and imaging agents for magnetic resonance imaging or computer tomography—was applied to increase therapeutic efficacy [77,189,190,191,192,193]. Examples include NPs with GPC-3 antibodies, which are abundant in PLC [154], and platelet-loaded levantinib/sorafenib that adheres to tumor blood vessels and tumor-circulating cells, therefore increasing accumulation at those sites [5,194].

### 4.1. Targeting

Recent advancements in NP-based DDSs have shown significant promise in enhancing sorafenib efficacy for PLC. Targeting strategies, including GPC3, ASGPR1, LDL receptors, and GLUT1 transporters, improve cellular uptake, therapeutic efficacy, and tumor-specific accumulation. This section highlights diverse approaches employing functionalized NPs, liposomes, and polymeric carriers to deliver sorafenib, demonstrating improved clinical outcomes, reduced adverse effects, and synergistic therapeutic potential in preclinical and clinical studies.

#### 4.1.1. Antibodies

**Figure 9:**
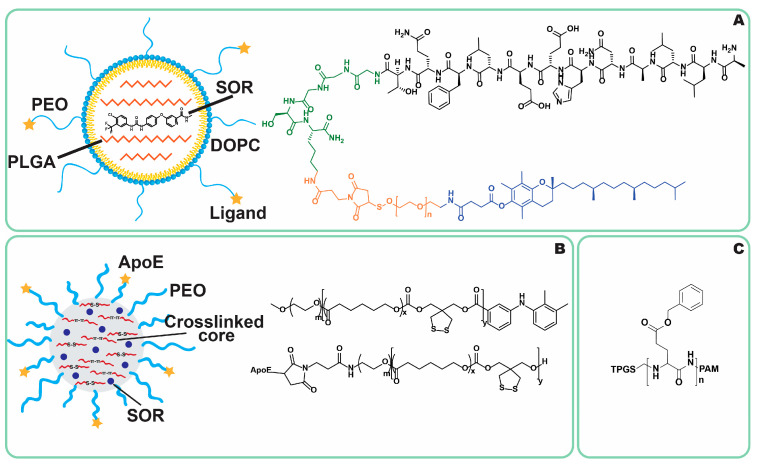
Structure of DDS prepared by (**A**) Feng et al. [106]; (**B**) DDS prepared by Li et al. [80]; (**C**) structure of compound prepared by Li et al. [195]. DOPC—dipalmitoylphosphatidylcholine; PLGA—poly(lactic-co-glycolic acid); SOR—sorafenib; PEO—poly(ethylene oxide); ApoE—apolipoprotein E; PAM—poly(amidoamine).

Su et al. [154] reported targeted polycaprolactone (PCL) polymeric NPs loaded with sorafenib that demonstrated improved clinical outcomes in human studies. These NPs were equipped with a tailored antibody designed to specifically bind to GPC3, a protein overexpressed in PLC cells. The DDS was administered via TACE and compared to standard therapy. Patients who received both the standard therapy and the DDS showed a higher disease control rate and lower incidence of adverse reactions compared to those who received only the standard therapy. Furthermore, the novel treatment was confirmed to be safe [154]. Similarly, Shen et al. [155] also targeted GPC3 for PLC treatment using sorafenib-loaded polymeric NPs. These NPs were composed of a diblock copolymer of PLGA and PEO modified with maleimide, functionalized with the hGC33 antibody through thioether bonds. The DDS was selectively internalized by GPC3-overexpressing HepG2 cells, but not by Li-7 cells, which lack significant GPC3 expression. This specificity was reflected in vitro, where HepG2 cell proliferation was strongly inhibited by the antibody-functionalized NPs, even without sorafenib, while Li-7 cells showed no inhibition. When sorafenib was encapsulated in the functionalized NPs, a synergistic effect was observed, leading to further suppression of HCC cell migration. In vivo studies using mice implanted with HepG2 or Huh7 cells revealed that the sorafenib-loaded, antibody-functionalized NPs had the highest potential for tumor growth inhibition. Tumor volumes in these mice were significantly smaller compared to other groups treated with naked antibody, sorafenib, non-functionalized NPs, or NPs without sorafenib. At the end of the treatment, the tumor volumes in mice treated with the targeted NPs were reduced by factors of 2 and 1.4 in HepG2 and Huh7 models, respectively, compared to the free sorafenib group [155]. Another study, by Feng et al. [106], focused on GPC3 receptor targeting. The authors developed a sophisticated nanomaterial consisting of a PLGA core encapsulating sorafenib, a DOPC shell, and a surface-functionalized PEO-modified D-α-tocopheryl succinate, capped with a GPC3-targeting peptide ligand (Figure 9A). The nanocarrier effectively targeted GPC3-positive PLC cells, such as Hep3B, while showing negligible uptake in GPC3-negative cells such as SK-Hep-1. Compared to NPs functionalized with alternative peptides, these GPC3-targeted NPs demonstrated superior uptake, leading to enhanced cytotoxicity in the GPC3-positive cells. In vivo evaluation using mice bearing human HCC xenografts confirmed that these targeted NPs preferentially accumulated in tumors composed of GPC3-positive cells. Fluorescence intensity, used as an indicator of NP presence, was three times higher in tumor tissue compared to other organs and persisted for an extended duration. Tumor growth studies further demonstrated that GPC3-targeted NPs exhibited the strongest tumor growth inhibition among all tested formulations [106]. These findings align with other studies mentioned in this section, underscoring the potential of GPC3-targeted approaches for PLC treatment [106,154,155].

Antibodies were also applied by Ye et al. [196] to enhance the delivery of sorafenib using liposomes. The liposomal formulation consisted of sorafenib, cholesterol, PEO–distearyl phosphatidylethanolamine, and chitosan cetyl quaternary ammonium salt, with the targeting moiety chemically bound to the liposome surface. In vitro studies on Huh-7 cells revealed a significantly lower survival rate for cells treated with the targeted liposomes than free sorafenib—18% versus over 70% after 72 h. A similar toxic effect was observed on HUVEC cells (human umbilical vein endothelial cells), highlighting the DDS’s potential to inhibit angiogenesis. In a PLC mouse model, the DDS demonstrated superior therapeutic outcomes, including slower tumor growth and reduced expression of sorafenib-related target proteins, compared to free sorafenib. Notably, liposomes without the targeting antibody showed negligible therapeutic improvement over free sorafenib, underlining the critical role of the targeting moiety [196].

#### 4.1.2. ASGPR

ASGPR is another ligand that was explored for PLC targeting. The authors used polysaccharides, which played a dual role as matrix and ligand, and simultaneously simplified preparation and cost [127], while others focused on chemical modification of the matrix with monosaccharides. The modified materials were lipids [197] and metal–organic frameworks (MOFs) [79]. Preclinical data on ASGPR are promising, showing increased accumulation and uptake by tumors [127], together with better treatment outcomes [127], marked by lower progression and suppression of metastases. For SLPNPs [79], N-acetylgalactosamine shifted its accumulation from the lungs to the liver. Non-targeted NPs were as effective as free sorafenib, but targeted NPs showed superior efficacy [197]. This highlights the ligand’s critical role in targeting. However, the heterogeneity of ASGPR expression between PLC cells was shown, which indicates that only some patients suffering from the disease could be treated in this way. Chirayil and Kumar [127] utilized pullulan both as an NP matrix and as a ligand for ASGPR1 targeting. To enhance its hydrophobicity and self-assembly properties, pullulan was conjugated with stearic acid via succinic and ethylenediamine linkers. This modification allowed the polymer to form micelles capable of encapsulating sorafenib. While empty NPs exhibited low cytotoxicity against PLC/PRF/5 cells, the sorafenib-loaded NPs showed significantly enhanced cytotoxicity, surpassing the efficacy of free sorafenib. This improvement was attributed to the interaction of the NPs with ASGPR1, promoting cellular uptake via the endocytic pathway and aiding in the evasion of efflux pumps. In vivo studies in mice demonstrated that the NPs facilitated more significant liver accumulation and prolonged retention of sorafenib compared to the free drug [127]. Tunki et al. [197] focused on galactose-functionalized solid-lipid NPs (SLNPs) for targeted delivery of sorafenib. Galactose was modified with H_2_N-PEO-NH_2_ and conjugated to the carboxylic groups present in SLNPs containing sorafenib. In vitro, the galactosylated SLNPs achieved the lowest IC_50_ values against HepG2 cells, whereas free sorafenib and non-galactosylated NPs showed comparable cytotoxicity. In vivo, oral administration of the galactosylated SLNPs in mice resulted in lower clearance and preferential liver accumulation. In contrast, non-galactosylated NPs, consistent with the natural biodistribution tendency of SLNPs, accumulated in the lungs [197]. These findings reinforce the potential of ASGRP1 targeting through galactose for PLC treatment. Galactose derivatives, such as N-acetylgalactosamine, are also promising ligands for ASGRP1 targeting. Hu et al. [79] developed a zinc-based MOF loaded with sorafenib and glucose oxidase, along with a tracer—rhodamine B—linked to N-acetylgalactosamine as the targeting ligand. The NPs demonstrated selective uptake by ASGPR-positive cells, such as HepG2 and Huh7, but not by ASGPR-negative HEK293 cells. In vitro, the NPs reduced cellular migration, suppressed VEGFR2 expression, and lowered cell survival rates. In vivo studies on tumor-bearing mice showed that the NPs predominantly accumulated in tumor tissues, inhibited tumor progression, and reduced lung metastases. The combination of glucose oxidase, which restricted glucose supply and starved cancer cells, with sorafenib enhanced the therapeutic outcomes.

#### 4.1.3. Apolipoproteins

Lipid-based NPs were explored in other combinations, e.g., with iron oxide for magnetic guidance or with Apos to target lipoprotein receptors. The last approach was also tested with an engineered polymeric matrix. The addition of Apos increased tumor, liver, and spleen accumulation compared to non-targeted NPs and showed better outcomes in vivo on mice [80,136]. Another study focused on the development of magnetic NPs and the proper application of a magnetic field [198]. The magnetic guidance is challenging in vivo, as there is an external magnetic field that can organize NPs in 2D space but not in 3D space and is limited in terms of deeper parts of the body. Moreover, the effect of accumulation is often disturbed once the field is removed, meaning that patients must have been exposed to it for a longer period of time [199,200,201]. Solid-lipid NPs (SLNPs) were investigated by Iacobazzi et al. [198] for sorafenib delivery. These NPs were coated with PEO and loaded with superparamagnetic iron oxide NPs, imparting paramagnetic properties. In vitro studies revealed that applying a magnetic field significantly increased NP uptake and intracellular iron content in HepG2 cells compared to free sorafenib or non-targeted NPs. In vivo optimization of magnetic field application in mice demonstrated that using two separate magnets was more effective for liver-specific accumulation of both sorafenib and iron than a single magnet setup [198]. Wang et al. [136] employed lipid-based NPs for the co-delivery of sorafenib and dihydroartemisinin, with an ApoB coating to target LDLRs. This DDS showed the greatest reduction in HepG2 cell viability compared to free drugs, single-drug NPs, or non-coated NPs. Enhanced therapeutic effects were attributed to targeting capabilities, as ApoB-100-coated NPs exhibited greater cellular internalization. In mice, the DDS significantly reduced tumor volumes, yielding approximately two- and threefold smaller tumors compared to non-coated NPs and free sorafenib, respectively, highlighting its therapeutic potential [136]. Li et al. [80] synthesized a polymeric material (Figure 9B) functionalized with either ApoE or mefenamate, an anti-inflammatory and analgesic agent. The material self-assembled into micelles approximately 40 nm in diameter, featuring a disulfide-crosslinked core containing sorafenib and a PEO outer shell terminated with ApoE. The authors investigated the relationship between ApoE surface density and antitumor efficacy, concluding that a 7.5% ApoE density was optimal. Both lower and higher densities led to reduced antitumor effects. The presence of ApoE on the micelle surface was crucial for enhancing cellular uptake, as micelles without ApoE exhibited ninefold lower uptake in SMMC-7721 cells. Furthermore, in vivo biodistribution studies demonstrated effective tumor targeting, with ex vivo fluorescence intensity in tumors being nearly three times higher in the group treated with ApoE-modified micelles. In orthotopic SMMC-7721 tumor models, the DDS showed superior therapeutic performance, evidenced by smaller tumor volumes, reduced blood vessel density, and increased survival rates. These results underline the advanced pharmaceutical properties of this targeted DDS compared to non-targeted therapies [80].

#### 4.1.4. GLUT and CD44

Alternative to ASGPRs are GLUT receptors and CD44. For both receptors, ligands are also saccharides. Glucosamine-modified and hyaluronic-based NPs revealed inhibition of metastasis, with slower growth of primary tumor [81,133]. Meng et al. [133] exploited overexpressed GLUT1 transporters in PLC cells by grafting glucosamine, a ligand for GLUT1, onto the surface of PEO-disulfide–PCL micelles encapsulating sorafenib. The micelles exhibited a tumor-responsive release mechanism due to the GSH-enriched environment, enhancing drug release. This DDS demonstrated significantly higher uptake by HepG2 cells when functionalized with glucosamine, leading to superior anticancer effects. The targeted micelles revealed the lowest IC_50_ values—around three times lower than free sorafenib—and exhibited enhanced inhibition of cancer migration. In vivo, glucosamine-modified micelles showed the greatest therapeutic efficacy, achieving the smallest tumor volumes (~250 mm^3^ compared to ~1000 mm^3^ with free sorafenib) [133]. Ma et al. [81] developed dendronized hyaluronic acid-based micelles to co-deliver sorafenib and paclitaxel to PLC cells by utilizing CD44-mediated uptake. This strategy significantly enhanced the intracellular levels of the delivered drugs. In vitro studies demonstrated that HepG2 cells exhibited the lowest relative migration ability and cell viability when treated with the micelles containing both sorafenib and paclitaxel. The DDS was further evaluated in vivo using Heps tumor-bearing mice, where it showed superior tumor growth inhibition compared to micelles without hyaluronic acid or a simple mixture of the two drugs [81].

#### 4.1.5. Folate Receptor

Folic acid receptor overexpression is uncommon in PLC. However, several cell lines are exhibiting it [146], and it was successively exploited to obtain better therapeutic results using folic acid-modified NPs. Zhang et al. [98] developed bismuth-based mesoporous nanomaterials loaded with sorafenib and coated with a PEO–folic acid conjugate. This DDS was tested against HCC in combination with radiotherapy. It turned out that the DDS in combination with X-ray exposure (6 Gy) decreased the viability of SMCC-7721 and BEL-7402 cells, compared to sorafenib or X-ray treatment only. In vivo, the therapy demonstrated superior outcomes with approximately four and two times the tumor volume reduction in comparison to the results obtained by sorafenib or radiotherapy only, respectively. Additionally, the nanomaterials effectively accumulated in tumor tissues, enabling CT visualization and demonstrating their theranostic capabilities [98]. Wang et al. [99] covalently linked folic acid to bovine serum albumin (BSA) and incorporated it into BSA NPs loaded with sorafenib. The targeted NPs demonstrated significantly higher cellular uptake by SMMC-7721 cells (2–6 times more than non-targeted counterparts) and increased drug accumulation in the liver, achieving levels 24 times higher than free sorafenib. While sorafenib concentrations in the liver were statistically similar between targeted and non-targeted NPs, the targeted formulation notably elevated drug levels at the tumor site, improving therapeutic outcomes [99].

#### 4.1.6. Vitamin E

Vitamin E and its derivatives are also molecules proposed for the targeting of PLC cells. This approach was employed by Li et al. [195], who utilized D-α-tocopheryl polyethylene glycol succinate (TPGS) conjugated to poly(amidoamine) dendrimers (Figure 9C) to prepare NPs loaded with sorafenib. Incorporating TPGS improved cellular uptake in HepG2 cells, achieving an uptake efficiency of 75.5%, compared to 42.7% for PEO-based NPs. Additionally, the TPGS-modified NPs demonstrated higher cytotoxicity toward HepG2 cells, with an IC50 of 0.75 μg/mL, compared to 6.8 μg/mL for free sorafenib and 9.5 μg/mL for PEO-based NPs. In vivo study of HepG2 xenograft-bearing nude mice further confirmed the therapeutic potential of TPGS-modified NPs, which exhibited the greatest tumor growth inhibition among all tested formulations [195].

#### 4.1.7. CXCR4

Last but not least is the approach of using peptides as ligands. Zheng et al. [111] developed polymeric NPs for the co-delivery of sorafenib and metapristone, composed of a block copolymer of PLGA and PEO, functionalized with the LFC131 peptide targeting CXCR4. The combination of LFC131-NPs loaded with sorafenib and metapristone at a drug molar ratio of 5:1 demonstrated the strongest proliferation inhibition of SMCC-7721 cells, significantly outperforming free drugs and almost completely suppressing colony formation in vitro. The targeted NPs exhibited prolonged circulation, reduced premature release at blood pH, and preferential accumulation in malignant tissue. In a PLC mouse model, tumor volumes after 7 days of treatment with the combinatory targeted NPs were 1.3-fold and 2.0-fold smaller than those in mice treated with free sorafenib or metapristone, respectively [111].

The main outcomes of the targeted DDSs for PLC treatment combined with SOR or its derivatives are summarized in Table 3.

### 4.2. Bioengineering Approaches

**Figure 10:**
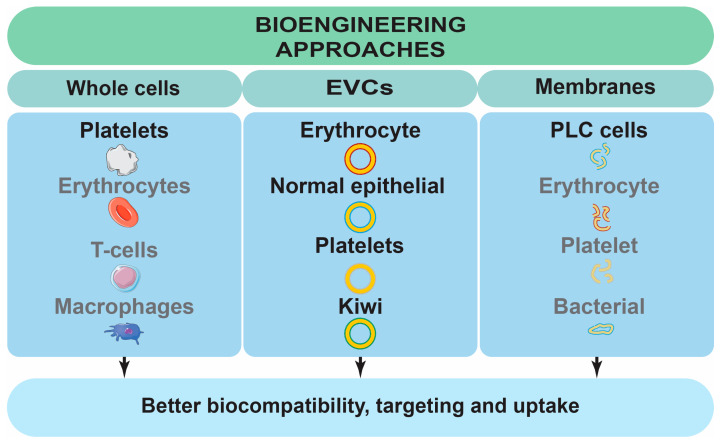
Bioengineering approaches for DDSs. Black font—applied for SOR and their derivatives, grey—not applied. Based on [209,210,211,212,213]. EVCs—extracellular vesicles.

One of the obstacles to using NPs is their biocompatibility. There are several approaches that increase it. Inorganic materials can be coated with organic ones that increase hydrophilicity and give a “stealthy” characteristic to the device [204]. The surface charge is also important, with a negative charge being preferred [205]. Another factor is biodegradability, and if this cannot be accomplished, then inert, easy-to-exert materials should be used to avoid accumulation [206]. Another interesting approach to improving biocompatibility utilizes biological structures, such as cells, extracellular vesicles, and biological membranes (Figure 10) [207,208].Exosomes represent promising vehicles for the delivery of anticancer drugs due to their excellent targeting capabilities and biocompatibility. In particular, exosomes derived from cancer cells exhibit superior targeting properties. However, their clinical application is significantly hindered by the presence of cancer-related or cancer-promoting signaling molecules, which pose a risk of exacerbating the disease [207].

Wu et al. [214] used a PLC cell membrane and coated it with polymeric (PAE–PEG–NH_2_) NPs with embedded lenvatinib. The membrane demonstrated homologous binding ability. It was achieved by CD-147, which is responsible for intercellular adhesion in the tumor. The coating reduced phagocytosis by immune cells and increased blood retention time. Moreover, the NPs were able to accumulate in tumor in vivo (mice). The authors also observed better therapeutic outcomes in the group where the coated NPs were administered compared to the non-coated ones or free drug [214].

Zhang et al. [208] enhanced the targeting properties of sorafenib by encapsulating it in extracellular vesicles derived from red blood cells. These vesicles were preferentially taken up by liver macrophages, leading to improved therapeutic outcomes in an HCC-LM3 orthotopic liver cancer model. Tumor size was reduced, and angiogenesis was more strongly inhibited compared to standard sorafenib therapy. Furthermore, systemic toxicity was lower, with no observed histopathological organ damage or skin thinning, though liver function impairment was slightly higher for this DDS compared to standard therapy [208]. On another hand, Tanaka et al. [194] employed platelets to deliver sorafenib or lenvatinib. They observed that platelet counts correlated with tumor volume and noted platelet adherence and activation in tumor blood vessels. Platelets collected from PLC rat models were loaded with the drugs and reintroduced to the donors. The carriers accumulated in tumors and surrounding tissues, causing more significant necrosis of malignant cells than free sorafenib, while toxicity levels remained similar [194]. Platelets were also used by Da et al. [5] for the construction of a biomimetic delivery platform for sorafenib. The drug was loaded into mesoporous silica and then coated with platelet membranes with immobilized programmed death ligand 1 antibody. The idea behind this was that circulating tumor cells form clusters with platelets’ defensive coating, which prevents the immune system and therapeutic agents from killing them. By coating with platelet membranes, the authors were able to target the clusters, as the study on mice with injected H22 cells (experimental metastatic model of PLC) revealed that the clusters were co-localized with the DDS, while bare particles were not. Moreover, the metastatic tumors in mice treated with the engineered platelets were infiltrated more by immune cells, such as CD8+ and CD4+ T cells, than in groups where sorafenib, antibody, or silica-based NPs were applied. The result also suggests the antimetastatic ability of the platelet membrane platform, as fewer metastatic nodules were found in the lungs [5]. He et al. [207] utilized exosomes from normal epithelial cells, equipping them with the HN3 antibody (targeting GPC3), sorafenib, and sgIQ 1.1 plasmids (encoding CRISPR/Cas9 for IQGAP1 silencing). These exosomes exhibited selective uptake by Huh7 cancer cells, with sevenfold lower uptake in LO2 cells (it should be noted that LO2 was misidentified as a human hepatocyte cell line [215]). The exosomes from normal cells were associated with a higher antiproliferative effect in vitro on Huh7 cells than those from cancer cells, as well as free sorafenib [207]. Exosomes are also a potential oral DDS for sorafenib. Fang et al. [78] prepared kiwi-derived exosomes loaded with sorafenib for oral administration. The vesicles were stable under gastrointestinal conditions and permeated more through Caco-2 monolayers (study to test penetration ability through intestinal epithelial barriers) than free sorafenib. The natural lipid composition of the exosome membrane (phosphatidylcholine) promoted the DDS transfer from the intestines to the liver and stayed mostly in the mouse organ. In addition, the vesicles were more willingly uptaken by HepG2 cells and did not negatively affect sorafenib effectivity, but the effect was slower. The liver targeting and accumulation might also be caused by digalactosyl diacylglycerols, which were identified in the composition by the authors [78], and galactose is a known ligand for ASGPR1 [126]. Therefore, the DDS is an interesting candidate for further in vivo examination.

The main outcomes of the bioengineered DDSs for PLC treatment combined with SOR or its derivatives are summarized in Table 4.

### 4.3. Combinatory NPs

**Figure 11:**
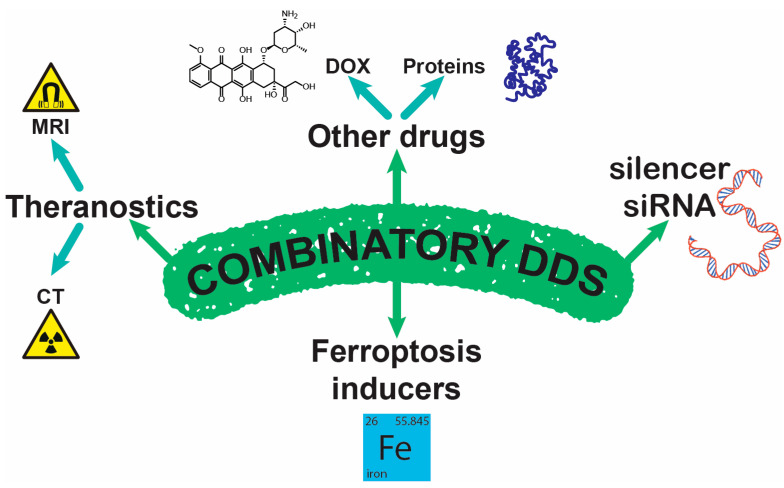
Various approaches used in combinatory NPs. MRI—magnetic resonance imaging; CT—computer tomography; DOX—doxorubicin.

This chapter explores recent advancements in combinatory NP-based DDSs for PLC treatment, focusing on strategies that integrate multiple therapeutic agents for enhanced efficacy. Various DDS designs utilize functionalized NPs, polymeric micelles, and microspheres to overcome barriers in PLC therapy. The incorporation of targeting ligands, tumor microenvironment-responsive materials, and synergistic drug combinations is discussed (Figure 11). Examples include SP94-functionalized NPs [77], magnetically triggered release systems [189], and formulations designed for dual delivery of chemotherapeutic agents [190] and siRNA [77]. These innovations collectively represent a significant step forward in precision oncology, demonstrating how combinatory NPs can redefine HCC treatment standards.

#### 4.3.1. Theranostics

Theranostic approaches allow for simultaneous treatment and diagnosis of patients. Iron and its oxides in the DDS formulations have been common contrast agents for MRI and CT. Polymer-based NPs—PLGA, pluronic, and PCL—were mostly used and enriched with freshly prepared Fe_3_O_4_. The use of pluronic was exceptional because, in such a case, fluorescent indocyanine was applied. These nanodevices demonstrated superior therapeutic outcomes by enhancing tumor targeting, reducing hypoxia, and enabling theranostic applications. Moreover, significant inhibition in tumor growth, improved drug release efficiency, and the induction of higher cancer cell apoptosis compared to standard treatments were noted. The data demonstrate the development of theranostics in HCC [189,190,191,192,193]. Park et al. [190] investigated the efficacy of sorafenib-loaded microspheres in a preclinical rat HCC model using TAE. The DDS was composed of PLGA microspheres containing Fe_3_O_4_ NPs, which also functioned as MRI contrast agents. The microspheres were primarily deposited in well-vascularized regions of the tumor and normal liver tissue without inducing liver toxicity. In contrast, the therapeutic impact on HCC was significant. The group developing the DDS combined with the doxorubicin–lipiodol emulsion [190]—the golden standard for HCC therapy [191]—showed superior tumor growth inhibition compared to the group treated with standard therapy alone. Additionally, the DDS group exhibited more significant reductions in microvessel development and increased apoptosis of HCC cells [190]. Li et al. [192] further evaluated the effectiveness of TACE using PLGA microspheres loaded with sorafenib and catalase in a rabbit VX2 liver tumor model. This DDS demonstrated enhanced embolization of blood vessels and better blockage of tumor blood supply compared to other tested alternatives. Catalase played a pivotal role in reducing tumor microenvironmental hypoxia by decomposing hydrogen peroxide into oxygen and water. This alleviation of hypoxia suppressed the expression of PD-L1, a key protein in tumor immune evasion, and facilitated CD8+ T cell migration. Consequently, a greater extent of liver cancer cell necrosis was observed, and tumor volume significantly decreased compared to pre-treatment levels. Among all tested microsphere formulations, the combination of sorafenib and catalase showed the most pronounced therapeutic effects [192]. Wu et al. [193] created a nanoconstruct by stacking sorafenib and indocyanine via π–π interactions and coating it with pluronic. The device had theranostic capabilities, as indocyanine exhibited fluorescence under near-infrared irradiation. In vitro studies on Huh7 cells showed that this construct achieved superior cancer cell killing to the free combination of sorafenib and indocyanine, an effect further enhanced by NIR irradiation due to synergistic reactive oxygen species (ROS) generation. The therapeutic potential of the nanoconstruct was diminished with ROS scavengers, confirming the primary role of ROS in its mechanism. In vivo, the fluorescence allowed monitoring of tumor accumulation, which progressively increased over 24 h while sparing other organs. At the end of the treatment, the tumor weight in the nanoconstruct group was threefold smaller than in the free combination group, demonstrating its high efficacy [193]. Magnetic-triggered drug release has also been explored in PLC therapy, particularly in TACE. Cho et al. [189] designed Janus particles composed of PCL and PLGA with embedded Fe_3_O_4_ nanocubes for the dual delivery of regorafenib and doxorubicin. When exposed to a non-static magnetic field, the particles underwent rotational motion, enhancing drug release. The optimal magnetic field frequency was identified as 100 MHz, which significantly improved HepG2 cell killing compared to groups not subjected to the magnetic field. In vivo, the DDS was administered to an orthotopic HCC rat model via transcatheter delivery. The particles accumulated at the tumor site, which was effectively visualized using magnetic resonance contrast properties of iron oxide, further confirming the precision of the magnetic-field-guided delivery system [189].

#### 4.3.2. Iron-Containing

Other important iron-enriched DDSs are those that are engineered to induce a special type of apoptosis called ferroptosis. This approach was also tested with peptides, which acted as ligands for specific receptors and drug transporter channel openers, or with siRNA, which silences ferroptosis suppression genes. It is worth noting that siRNA is often used by authors to sensitize PLC to sorafenib. Yue et al. [216] developed Prussian blue NPs composed of Fe^2+^ and Fe^3+^ ions coordinated with CN^−^ ions, entrapping sorafenib within their cubic structure and coating the NPs with chitosan. This dual-functional design enabled controlled sorafenib release while the degradation of the matrix released ferrous ions, disrupting the oxidant/antioxidant balance and triggering ferroptosis. In vitro, the vehicle demonstrated enhanced cancer cell killing at a reduced pH (6.5) and in the presence of H_2_O_2_. In vivo, after injection into mice, fluorescently labelled NPs accumulated at the tumor site, unlike the free label. Tumor volume in mice treated with the DDS was approximately twice as small as in those treated with free sorafenib or empty NPs, highlighting the superior therapeutic potential of this platform [216].

**Figure 12:**
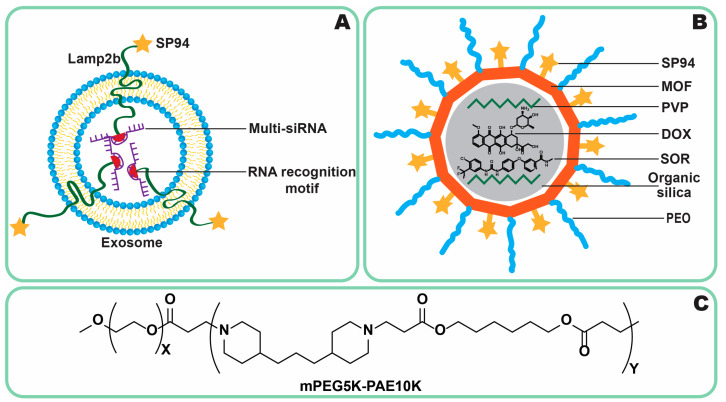
DDS prepared by (**A**) Li et al. [77]; (**B**) Ling et al. [165]; (**C**)—copolymer used as a matrix for DDS by Song et al. [218]. SP94—A tumor-targeting peptide; PEG—polyethylene glycol; PAE—poly(amino ester); DOX—doxorubicin; SOR—sorafenib; PVP—polyvinylpyrrolidone; MOF—metal-organic framework; PEO—poly(ethylene oxide).

Liu et al. [116] co-administered the iRGD peptide, which contains both a tumor-homing motif and a tissue-penetrating motif, to enhance the penetration of iron-based NP loaded with sorafenib into PLC tumors. This approach aimed to overcome therapeutic limitations posed by tumor structural barriers. Adding iRGD significantly increased intratumoral drug concentration both in vitro and in vivo by opening drug transport channels. Furthermore, the combination of sorafenib with iron-based NPs triggered the release of iron ions, inducing ferroptosis—a form of regulated cell death. This combination exhibited synergistic effects in eliminating H22 tumors in a mouse model. Co-treatment with iRGD further amplified these effects, surpassing the efficacy of sorafenib or iron-based NPs alone while minimizing toxicity [116]. A similar strategy was explored by Li et al. [77], which utilized SP94-modified exosomes (Figure 12A) for delivery of a siRNA that silences GPX4 and DHODH—genes responsible for the suppression of ferroptosis. In this approach, the exosomes were produced by transfection of HEK-293T cells with SP94-Lamp2b-RRM expressing plasmid and the siRNA. The produced protein, SP94-Lamp2b-RRM, consisted of the SP94 part that was responsible for targeting Lamp2b [77], which is a protein naturally found in exosome membrane [217], and RRM, an RNA recognition motif that increased exosomal loading with siRNA. The exosomes were then tested for whether they could sensitize HCC to sorafenib. As a result, in the in vivo model of the cancer, the combinatory therapy was more successful in prolonging survival time [77].

#### 4.3.3. Co-Delivery

NPs were also designed for co-delivering sorafenib and doxorubicin or other agents to enhance therapeutic outcomes in PLC. Proteins such as SIRT7 inhibitors and targeting ligands like SP94 were incorporated to improve drug sensitivity and tumor targeting, while polymeric and functionalized NP platforms enabled controlled, responsive drug release in the tumor microenvironment. Key findings include the superior efficacy of SOR and SIRT7 inhibitor-loaded NPs, achieving an eightfold tumor volume reduction compared to free drugs. Polymeric micelles co-delivering SOR and a PI3Kγ inhibitor extended the half-life of the drug and reduced tumor volume threefold. SP94-functionalized NPs loaded with SOR and DOX showed enhanced tumor targeting, achieving significantly lower tumor volumes than non-targeted systems. Zhang et al. [219] synthesized an amphiphilic polymer composed of PEO, a disulfide bridge, polylysine, and cholesterol, designed for the dual delivery of sorafenib and 2800Z—a protein inhibitor of SIRT7 that enhances chemosensitivity to sorafenib. The dual-loaded NPs significantly reduced the viability and colony formation of Huh7.5-luc cells compared to equivalent doses of free drugs. In vivo, these NPs demonstrated targeted tumor accumulation and achieved the most pronounced tumor growth suppression in mice treated with NPs containing sorafenib and 2800Z in a 1:1 mass ratio. Tumor volume in this group was approximately eight times smaller than in mice treated with the free drug combination, highlighting the superior efficacy of the DDS [219]. Li and Zhao [220] developed polymeric micelles based on PEO and hydroxyethyl starch to co-deliver sorafenib and a PI3Kγ inhibitor, TG100-115. Sorafenib was physically entrapped, while TG100-115 was chemically conjugated to the micelle matrix. The NPs exhibited pH- and enzyme-responsive drug release, mimicking the tumor microenvironment with enhanced sorafenib release in the presence of α-amylase. In vitro, these NPs showed the highest cell viability inhibition in Hep-3B cells compared to free drugs or blank micelles. In vivo, the NPs extended the half-life of both drugs and achieved a threefold more significant tumor volume reduction in PLC-bearing mice compared to the free drug combination [220]. Ling et al. [165] developed an SP94-functionalized NP (Figure 12B) (NP) for targeted PLC therapy. The NP consisted of an inner layer of PVP and an outer layer of a copper-based MOF. Two drugs, doxorubicin (DOX) and sorafenib (SOR), were loaded into the NP, and its surface was functionalized with SP94 for targeting and PEO for stability. The NP demonstrated pH- and GSH-sensitive degradation, ensuring accelerated drug release at the tumor site and after cellular uptake. In vivo studies using a xenograft HCC tumor model in nude mice revealed that the SP94-functionalized NPs accumulated effectively at the tumor site, while non-functionalized NPs showed significantly reduced tumor targeting. Tumor volume analysis further conformed the observations that SP94-targeted SOR/DOX NPs achieved substantially lower tumor volumes than non-targeted SOR/DOX NPs or free SOR/DOX. This highlighted the critical role of SP94 functionalization in enhancing therapeutic efficacy [165].

#### 4.3.4. siRNA

Other siRNAs that were used by authors are midkine-siRNA and Tim-3 targeting siRNA. Their role was to sensitize PLC to sorafenib through the downregulation of angiogenesis or an improvement in immune response. Co-delivering sorafenib and midkine-siRNA achieved 85% tumor growth inhibition and significant downregulation of HCC markers, overcoming drug resistance. Similarly, a polymer-based DDS for Tim-3 siRNA and sorafenib exhibited improved tumor targeting, immune response enhancement, and a 50% reduction in tumor volume compared to standard therapies [218,221]. Specifically, Younis et al. [221] utilized lipid-based NPs modified with PEO and SP94, a PLC-targeting peptide, for the co-delivery of sorafenib and midkine-siRNA to overcome HCC chemoresistance. The approach achieved an 85% tumor growth inhibition and an 80% silencing of the MK gene. This silencing was critical for overcoming resistance to sorafenib, as prolonged treatment of HepG2 cells with the free drug resulted in MK gene upregulation. Treating these resistant cells with the DDS reduced cell viability to ~50%, compared to ~95% with free sorafenib. Additionally, the DDS downregulated key HCC markers, including alpha-fetoprotein, osteopontin, and VEGF-1, further demonstrating its therapeutic potential [221]. Song et al. [218] utilized a PEO polymer grafted with a hydrophobic segment (Figure 12C) to co-deliver sorafenib and siRNA targeting Tim-3, a protein associated with HCC progression. The siRNA was efficiently released in acidic environments and taken up by HCC cells. In vitro studies on H22 cells demonstrated that the DDS had an IC_50_ value twice as low as the free combination of sorafenib and siRNA. In vivo, the DDS resulted in prolonged siRNA circulation and increased tumor accumulation. This enhanced delivery translated into improved antitumor effects in a mouse model of PLC, where tumor volume in the DDS group was reduced by half compared to the free drug combination group. Further analysis of tumor tissues revealed stronger immune responses and reduced blood vessel density in the DDS-treated group, underscoring its potential as an advanced treatment platform [218].

The main outcomes of the combinatory DDSs for PLC treatment combined with SOR or its derivatives are summarized in Table 5.

## 5. Conclusions

Unsurprisingly, NP and biomaterial-based drug delivery strategies for sorafenib have been intensively studied in PLC due to the promising effectiveness, albeit with some limitations, of the parent molecule. Being aware that current therapies face significant limitations due in part to chemoresistance, altered metabolism, and/or genetic modulations, the authors feel motivated to present the studies in this review.

**Figure 13:**
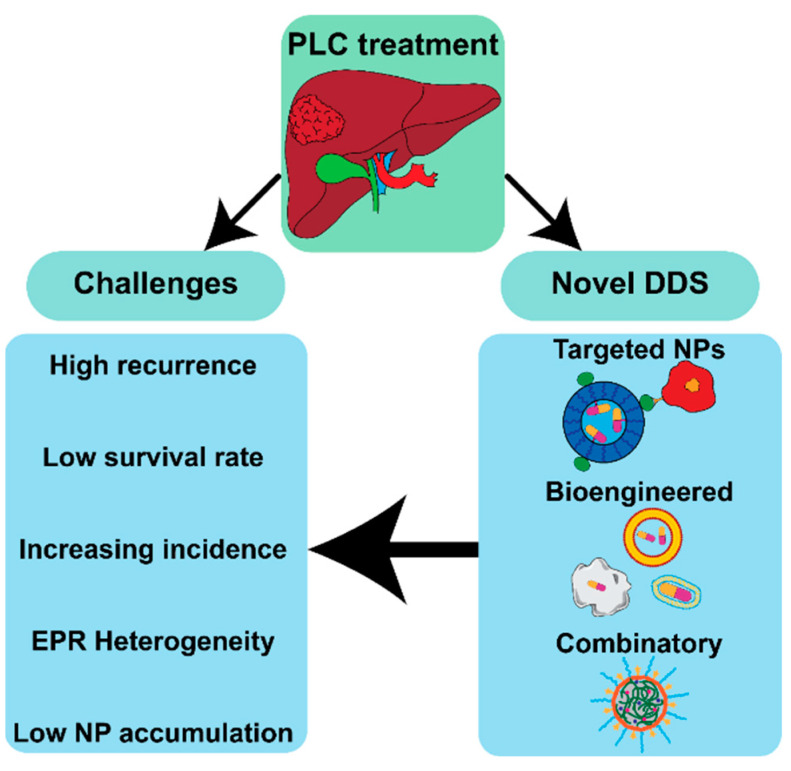
Novel DDSs address the challenges of PLC.

While there is no doubt that the scientific data regarding DDSs for the treatment of PLC indicates their usefulness, there is some uncertainty regarding the broader application of NPs and their related constructs within DDSs. The reason for it remaining contentious is that they are not always dedicated from the outset to a specific type of cancer cells. The critical issue lies in the reliance on the EPR effect for drug delivery in humans. This strategy is controversial due to significant differences between human cancers and preclinical animal models. In particular, human tumors often undergo accumulated mutations over a specific time before initiating growth, which results in more significant heterogeneity and a reduced presence of “leaky vasculature.” Consequently, challenges related to drug accumulation and permeation persist. Other factors such as high recurrence, lack of adjuvant chemotherapy, increasing incidence, and low accumulation of non-targeted NPs further decrease therapeutic outcomes. That said, in many studies, it was indicated that the clinical efficacy of these approaches was comparable to that of standard therapies [27,89,92,93]. A solution to these drawbacks would be a more advanced form of targeted bioengineered and combinatory NPs (Figure 13).

Current treatments for PLC, such as TACE, address these limitations by enhancing localized drug concentration at the tumor site [45,46]. TACE, especially when combined with drug-eluting microspheres or NPs, has demonstrated superior efficacy compared to standard therapies, achieving higher survival rates, lower recurrence rates, and improved disease control [154,187,188]. Another essential consideration is the preferential accumulation of NPs in the liver [77,78,79,80,81,82]. Strategies to enhance the transiting of NPs from the liver to the tumor have shown significant potential, e.g., Kupffer cells, which demonstrate higher uptake of NPs (~200 nm), exhibit tumor-homing capabilities, and thus migrate to the tumor site [83,84]. Alternatively, smaller NPs (6–12 nm) have the ability to accumulate in the liver and subsequently migrate intercellularly to the tumor [90,91]. These mechanisms present promising routes for improving drug delivery to tumors and enhancing therapeutic outcomes.

The targeted therapy of PLC holds the potential to overcome mechanisms of chemoresistance (MOCs) [94,95,96], with promising targets including GPC3 [154], GRP78 [77,165,221], ASGPR [79,127], GLUT1 [133], iRGD [116], and LDLR [80,136]. On the other hand, some explored targets, such as CD44 [81], CXCR4 [111], or FR [98,99], seem to have questionable utility due to low or nonspecific expression [108,146,147,183]. Targeted therapy is when there is the overexpression of specific markers in cancer cells, which then increases the ratio of drug delivered to tumors compared to healthy tissue. Although several studies have demonstrated the efficacy of this approach via enhanced drug delivery in reducing tumor volumes [106,116,127,155,195,197], clinical validation remains lacking. Notably, targeted delivery systems demonstrate approximately 1.5 times greater NP uptake in tumors than non-targeted systems, although overall tumor accumulation remains suboptimal, requiring the continued use of TACE to ensure sufficient local drug delivery [94]. Additionally, one MOC in PLC is associated with reduced drug uptake, linked to impaired SLCO receptor function. By targeting alternative overexpressed markers, targeted DDSs are capable of mitigating this issue [96]. Another potential application for targeted DDSs is in adjuvant chemotherapy, particularly considering the high recurrence and metastatic potential of PLC [222]. Developing effective adjuvant therapies will significantly improve long-term results.

Combination therapies could be considered another prospective strategy to address MOCs. As of 2024, sorafenib and its derivatives are co-administered with iron compounds, such as ferrous ions and oxides, to induce ferroptosis [77,116,216]. These combinations have also included proteins, RNA, or plasmids to silence or inactivate chemoresistance-associated pathways, leading to smaller tumor volumes and improved chemosensitivity [207,218,219,220,221].

Last but not least is the bioengineering approach for delivering drugs, which utilizes whole cells, their exosomes, or membranes. For example, platelets, known for their ability to adhere to tumoral vessels or circulating tumor cells, have been employed to enhance drug accumulation in tumor tissue, inhibit metastasis, and increase immune cell infiltration. This approach constitutes an interesting strategy for adjuvant therapy by targeting blood-circulating cancer cells [5,194]. Similarly, red blood cell vesicles are associated with higher uptake by Kupffer cells, improving liver-targeted delivery [208], while cancer exosomes can directly fuse with PLC cells to deliver therapeutic agents [77,214]. Even plant-derived exosomes, such as those from kiwi, have demonstrated uptake by PLC cells, likely due to the presence of galactose on their surfaces [78].

The discussed DDSs for PLC treatment highlights the efficiency of TACE combined with drug-eluting microspheres or NPs, which has shown higher survival rates, lower recurrence, and better disease control compared to standard therapies. However, challenges remain, particularly in drug accumulation and permeation in tumors due to limited “leaky vessels” in human cancers. NPs tend to accumulate in the liver, with mechanisms such as Kupffer cells or smaller NPs (6–12 nm) facilitating tumor migration. Targeted therapies focusing on overexpressed markers such as GPC3 or LDLR show better efficiency in animal models, but their clinical efficacy is still under investigation. Combining therapies (e.g., sorafenib derivatives with ferroptosis inducers or RNA/plasmids) has improved chemosensitivity and reduced tumor volume. Biomimetic approaches, using cells or exosomes (e.g., platelets or RBC vesicles), have shown better efficiency in animal models for tumor targeting, immune response enhancement, and metastasis prevention, particularly in adjuvant chemotherapy.

It seems that ongoing and future research regarding PLC should be focused on drug delivery systems to overcome the limitations of tumor heterogeneity, low drug accumulation, and a reduction in recurrence. By addressing these gaps in current treatments, synergistic combinations of existing therapies and advanced delivery methods could ultimately improve survival rates and patient conditions.

## Figures and Tables

**Table 1:** Factors influencing NP accumulation and uptake in PLC treatment.

Factor	Impact on NP	Effect on Drug Delivery	Ref.
Hydrophobic surface	Promotes binding of plasma protein and liver uptake	Higher accumulation in liver	[83,84,85,86]
Surface charge	Positively charged NPs → hepatocyte uptake; negatively charged NPs → Kupffer/endothelial uptake	Affects targeting efficiency and biodistribution	[87]
Size 150–200 nm	Enables crossing capillary fenestrations	Improves hepatocyte internalization	[88]
Size 12 nm	Optimal tumor accumulation via EPR effect	Minimizes nonspecific organ uptake	[90,91]
Size 6 nm	Rapid renal clearance	Reduces therapeutic utility	[90,91]
Kupfer cell uptake	Excretion into intestines	Limits drug delivery to tumor	[86]
Tumor-homing Kupffer cells	Migration to tumor tissues	Potential to enhance NP accumulation in tumors	[83,84]
Targeting moiety	Uses specific receptors on cancer cells	~1.5× higher delivery efficiency than non-targeted systems	[89,94]

**Table 2:** Summary of molecular targets for PLC treatment and diagnosis.

Target	Expression Pattern	Ligands	Challenges
Glypican-3	Highly expressed in HCC, minimal in healthy liver and other tissues [102] (except placenta [103])	L5 peptide, truncated L5 [104,105], recombinant human GPC3 core protein [106], antibodies, aptamers [107]	Limited ligand stability (peptides), potential off-target effects during pregnancy [103]
CXCR4	Overexpressed in PLC, also found in normal tissues (bone marrow, lymphoid) [108]	Peptides (LFC131), small molecules (AMD3100 [109,110,111], cordycepin [112])	Off-target effects, challenges in tumor specificity
Neuropilin-1 (NP-1)	Expressed in tumors and endothelial cells [113,114], debatable overexpression in PLC [115]	iRGD peptide [116,117,118]	Limited evidence in PLC, primarily studied in pancreatic cancer [119,120,121]
GRP78	Overexpressed in drug-resistant PLC [122]	Peptides (SP94 [123], RGD)	Peptide ligands increase opsonization [124]
ASGPR	Predominantly in liver and PLC [125]	Saccharides (galactose, N-acetylgalactosamine [126]), glycoproteins, polymers (pullulan [127], arabinogalactan [128])	Variable expression in PLC [129]
GLUT-1	Elevated in PLC, increased in hypoxia-induced chemoresistant cells [130,131]	Saccharides (glucose [132], glucosamine [133], N-acetylglucosamine [134])	Risk of BBB penetration, potential off-target effects [135]
LDLR	High in cancer and liver cells [136,137,138,139]	Apolipoproteins (Apo B, E) [80,136]	BBB permeability [140], incomplete understanding of liver targeting mechanisms [83,84], widely expressed among tissues [136,137,138,139]
CD44	Low to moderate in PLC, high in stem-like PLC cells [141,142,143,144] (SK-HEP-1 [145])	Hyaluronic acid [81,133]	Low baseline expression in PLC [145]
Folate receptor	Generally low in PLC [146,147], but overexpressed in specific cell lines (SMMC-7721, BEL-7402) [98,99,100]	Folic acid [148]	Limited PLC specificity [146,147], potential BBB penetration [149]

**Table 3:** Targeted DDSs for PLC treatment combined with SOR or its derivatives.

DDS	Outcome	Study Type	Ref.
Callispheres—PVA microspheres with SOR delivered during TACE	- Higher t_1/2_ - Reduced drug intake - Reduced tumor volume - Higher remission rate - Lower recurrence - Reduced disease progression	In vivo (humans), compared to standard TACE with sorafenib	[187]
Callispheres—PVA microspheres with regorafenib delivered during TACE	- Higher objective response rates - Higher disease control rates	In vivo (humans)	[188]
NPs loaded with SOR targeting GPC3 via antibody, delivered during TACE	- Higher disease control rate - Lower incidence of adverse reactions	In vivo (humans), compared to standard therapy	[154]
NPs functionalized with hGC33 antibody, targeting GPC3, loaded with SOR	- Inhibited HepG2 cell proliferation - Reduced tumor volume in HepG2 and Huh7 models by factors of 2 and 1.4, respectively	In vivo (mice)	[155]
PLGA-based NPs functionalized with GPC3-targeting peptide for SOR delivery	- Superior tumor growth inhibition - Enhanced accumulation in tumors composed of GPC3-positive cells - Stronger tumor growth inhibition compared to free drug	In vivo (mice)	[106]
Liposomal DDS with targeting antibody (anti-VEGFR) for SOR delivery	- Significantly lower survival rate in Huh-7 cells compared to free sorafenib - Slower tumor growth in mice compared to free sorafenib	In vivo (mice), in vitro (humans)	[196]
Polymeric NPs functionalized with LFC131 peptide targeting CXCR4, co-delivering SOR and metapristone	- Stronger proliferation inhibition in SMCC-7721 cells - Reduced tumor volumes in mice compared to free SOR or metapristone	In vivo (mice), in vitro (humans)	[111]
Pullulan-based NPs self-assembled with stearic acid, targeting ASGPR1 for SOR delivery	- Enhanced cytotoxicity in PLC cells - Greater liver accumulation and prolonged retention in vivo	In vivo (mice), in vitro (humans)	[127]
Galactose-functionalized SLNPs for SOR delivery	- Lower IC_50_ in HepG2 cells - Preferential liver accumulation in vivo - Shift from accumulation of SLNPs from lungs to liver	In vivo (mice), in vitro (humans)	[197]
Zinc-based MOF loaded with SOR glucose oxidase and rhodamine B with N-acetylgalactosamine targeting ligand	- Selective uptake by ASGPR-positive cells (HepG2, Huh7) - Reduced cellular migration - Suppressed VEGFR2 expression - Inhibited tumor progression and reduced lung metastases in vivo	In vivo (mice) and in vitro (humans)	[79]
SLNP coated with PEO and loaded with SOR and iron oxide NPs	- Increased NP uptake and intracellular iron content in HepG2 cells under magnetic field - Enhanced liver accumulation in vivo	In vivo (mice) and in vitro (humans)	[198]
Bismuth-based mesoporous nanomaterials loaded with SOR and coated with PEO-folic acid conjugate	- Decreased viability of SMCC-7721 and BEL-7402 cells when combined with X-ray exposure (6 Gy) - Superior tumor growth inhibition compared to sorafenib or radiotherapy alone - Tumor volumes 4× and 2× smaller than sorafenib or radiotherapy alone, respectively - Effective tumor accumulation with CT visualization	In vivo (mice) and in vitro (humans), compared to sorafenib or radiotherapy alone	[98]
Lipid-based NPs co-delivering SOR and dihydroartemisinin with ApoB-100 coating targeting LDLRs	- The greatest reduction in HepG2 cell viability compared to free drugs, single-drug NPs, or non-coated NPs - Enhanced therapeutic effects due to ApoB-100 targeting - Greater cellular internalization - Tumor volumes 2× and 3× smaller than non-coated NPs and free sorafenib, respectively	In vivo (mice) and in vitro (humans), compared to free drugs, single-drug NPs, or non-coated NPs	[136]
BSA NPs loaded with SOR and folic acid	- 2–6× higher cellular uptake by SMMC-7721 cells compared to non-targeted NPs - 24× higher drug accumulation in the liver compared to free sorafenib - Elevated drug levels at the tumor site, improving therapeutic outcomes	In vivo (mice) and in vitro (humans), compared to non-targeted NPs	[99]
Glucosamine-functionalized PEO-disulfide–PCL micelles for SOR delivery	- Significant tumor uptake - Enhanced anticancer effects with significantly lower IC_50_ values	In vivo (mice), in vitro (humans)	[133]
HA-based dendronized micelles co-delivering SOR and PCX	- Enhanced intracellular drug levels - Superior tumor growth inhibition in mice	In vivo (mice), in vitro (humans)	[81]
TPGS-modified dendrimers for SOR delivery	- Enhanced cellular uptake - Greater tumor growth inhibition in vivo	In vivo (mice), in vitro (humans)	[195]
Polymeric micelles with ApoE or mefenamate, containing SOR	- Superior therapeutic performance in vivo - Increased cellular uptake and tumor targeting with ApoE modification	In vivo (mice)	[80]
Regorafenib-loaded PLGA microspheres delivered during TACE	- Sustained release for over 30 days - Antagonized miriplatin resistance in HepG2 cells - Improved anti-tumor efficacy when combined with miriplatin	In vivo (mice), in vitro	[202]
Regorafenib–gold NP conjugates	- Reduced toxicity to non-cancerous L929 cells - Superior effects on cell proliferation compared to free regorafenib	In vitro	[203]

Abbreviations: ASGPR1—asialoglycoprotein receptor 1; ApoB-100—apolipoprotein B-100; ApoE—apolipoprotein E; BEL-7402—a human liver cancer cell line; BSA—bovine serum albumin; CT—computed tomography; CXCR4—C-X-C motif chemokine receptor 4; DDS—drug delivery system; HA—hyaluronic acid; hGC33—anti-glypican 3 antibody; LDLRs—low-density lipoprotein receptors; LFC131—CXCR4 antagonist peptide; MOF—metal–organic framework; NPs—nanoparticles; PCL—polycaprolactone; PD-L1—programmed death ligand 1; PEO—poly(ethylene oxide); PLC—primary liver cancer; PLGA—poly(lactic-co-glycolic acid); SLNPs—solid lipid nanoparticles; SMCC-7721—a human liver cancer cell line; SOR—sorafenib; TPGS—tocopheryl polyethylene glycol succinate; TACE—transarterial chemoembolization; VEGFR—vascular endothelial growth factor receptor.

**Table 4:** Bioengineered DDSs for PLC treatment combined with SOR or its derivatives.

DDS	Outcome	Study Type	Ref.
Extracellular vesicles derived from red blood cells for SOR delivery	- Preferential uptake by liver macrophages - Tumor size reduction - Stronger angiogenesis inhibition - Lower systemic toxicity	In vivo (mice) and in vitro (cells), compared to standard sorafenib therapy	[208]
Platelets as carriers for SOR/lenvatinib	- Tumor volume correlated with platelet count - Platelet adherence and activation in tumor blood vessels - More significant necrosis of malignant cells compared to free drugs	In vivo (rats), compared to free sorafenib	[194]
Platelet-coated mesoporous silica NPs with PD-L1 antibody for SOR delivery	- Platelet clusters with PLC cells inside co-localized with DDS - Increased infiltration of immune cells (CD8+ and CD4+ T cells) - Fewer metastatic nodules in lungs	In vivo (mice), compared to non-coated NPs	[5]
Exosomes from normal epithelial cells with HN3 antibody for SOR delivery	- Selective uptake by Huh7 cancer cells - Higher antiproliferative effect in vitro compared to free sorafenib	In vitro (cells), compared to exosomes from cancer cells	[207]
Kiwi-derived exosomes for oral delivery of SOR	- Stable under gastrointestinal conditions - Enhanced uptake by HepG2 cells - Liver targeting and accumulation	In vivo (mice) and in vitro (cells), compared to free sorafenib	[78]
PAE–PEG–NH2 NPs coated with biological membranes from PLC cells (SMMC-7721) for lenvatinib delivery	- Reduced phagocyte uptake - Prolonged circulation time - No hemolytic activity - Preferential accumulation at the tumor site - Selective- fusion with cancer cells - Higher cytotoxicity compared to free lenvatinib	In vitro (human cells), In vivo (mice)	[214]

Abbreviations: CD4+—cluster of differentiation 4 (T helper cells); CD8+—cluster of differentiation 8 (cytotoxic T cells); DDS—drug delivery system; HN3—human antibody targeting GPC3; NPs—nanoparticles; PD-L1—programmed death ligand 1; PLC—primary liver cancer; SOR—sorafenib.

**Table 5:** Combinatory DDSs for PLC treatment combined with SOR or its derivatives.

DDS	Outcome	Study Type	Ref.
Sorafenib-loaded PLGA microspheres with Fe_3_O_4_ NPs (MRI contrast agents)	- Deposition in tumor and liver tissues - No liver toxicity - Higher tumor growth inhibition with doxorubicin–lipiodol emulsion	In vitro, in vivo (rat model)	[190]
PLGA microspheres loaded with sorafenib and catalase	- Enhanced embolization and tumor blood supply blockage - Reduced tumor hypoxia - Increased immune cell migration - Higher liver cancer cell necrosis	In vivo (rabbit VX2 liver tumor model)	[192]
Iron-based NPs loaded with sorafenib and iRGD peptide	- Increased drug penetration into tumors - Induced ferroptosis - Synergistic effect in tumor elimination - Higher intratumoral drug concentration	In vitro (cells), in vivo (mouse model)	[116]
SP94-modified exosomes for delivery of siRNA targeting GPX4 and DHODH with SOR co-treatment	- Enhanced ferroptosis sensitization - Prolonged survival time in vivo - Increased therapeutic efficacy in HCC	In vivo (mouse model)	[77]
SP94-functionalized NPs (copper-based MOF) with SOR and DOX	- Effective tumor accumulation - Greater tumor volume reduction - pH- and GSH-sensitive degradation - Accelerated drug release at tumor site	In vivo (xenograft HCC tumor model)	[165]
Amphiphilic polymer NPs with SOR (PEO, polylysine, cholesterol) with 2800Z	- Significantly reduced cell viability - Targeted tumor accumulation - Higher tumor volume suppression	In vitro (cells), in vivo (mouse model)	[219]
Polymeric micelles based on PEO and hydroxyethyl starch for delivery of SOR and TG100-115	- Enhanced sorafenib release in tumor microenvironment - Higher cell viability inhibition - Three times more significant tumor volume reduction	In vitro (cells), in vivo (PLC-bearing mice)	[220]
Lipid-based NPs modified with PEO and SP94 for delivery of SOR + midkine-siRNA	- 85% tumor growth inhibition achieved - 80% silencing of MK gene - Reduced cell viability in HCC chemo-resistant cells	In vitro (cells), in vivo (mouse model)	[221]
Prussian blue NPs (Fe^2+^, Fe^3+^, CN^−^) coated with chitosan for delivery of SOR	- Controlled sorafenib release - Triggered ferroptosis - Enhanced cancer cell killing at lower pH and in the presence of H_2_O_2_	In vitro (cells), in vivo (mice)	[216]
Nanoconstruct (sorafenib and indocyanine via π–π interaction, coated with pluronic)	- Higher cancer cell killing under NIR irradiation - Tumor accumulation - Threefold smaller tumor weight compared to free drugs	In vitro (Huh7 cells), in vivo (mice)	[193]
PEO polymer-based NPs for co-delivery of SOR and siRNA targeting Tim-3	- Tumor volume reduced by half compared to free formulation - Enhanced immune responses - Reduced blood vessel density	In vivo (PLC mouse model)	[218]
Janus particles for delivery of regorafenib and DOX (polycaprolactone, PLGA, embedded Fe_3_O_4_ nanocubes) for TACE	- Enhanced drug release with magnetic field exposure - Higher tumor killing with magnetic guidance - Effective tumor visualization with MRI contrast	In vitro (HepG2 cells), in vivo (HCC rat model)	[189]

Abbreviations: CD4+—cluster of differentiation 4 (T helper cells); CD8+—cluster of differentiation 8 (cytotoxic T cells); DDS—drug delivery system; HN3—human antibody targeting GPC3; NPs—nanoparticles; PD-L1—programmed death ligand 1; PLC—primary liver cancer; SOR—sorafenib.

## Data Availability

No new data were created or analyzed in this study. Data sharing is not applicable to this article.

## Abbreviations

The following abbreviations are used in this manuscript:

Apos	Apolipoproteins
ASGPR1	Asialoglycoprotein receptor 1
BBB	Blood–brain barrier
BSA	Bovine serum albumin
CD44	A cell-surface glycoprotein involved in cell–cell interactions
CT	Computer tomography
CXCR4	C-X-C motif chemokine receptor 4
DDS	Drug delivery system
FR	Folate receptor
GLUT-1	Glucose transporter 1
GPC-3	Glypican-3
GRP78	78 kDa glucose-regulated protein, heat shock protein
HA	Hyaluronic acid
HCC	Hepatocellular carcinoma
iRGD	Cyclic peptide, ligand of neuropilin-1
LDLR	Low-density lipoprotein receptor
MRI	Magnetic resonance imaging
NP	Nanoparticle
NP-1	Neuropilin-1
PCL	Polycaprolactone
PEO	Poly(ethylene oxide)
PLC	Primary liver cancer
PLGA	Poly(lactic-co-glycolic) acid
PVA	Poly(vinyl alcohol)
RGD	Arginine–glycine–aspartic
ROS	Reactive oxygen species
SI	Selectivity index
SOR	Sorafenib
SP94	Peptide targeting GRP78
TACE	Transarterial chemoembolization
TAE	Transarterial embolization
TPGS	Tocopherol polyethylene glycol succinate
